# Dynamic genetic differentiation drives the widespread structural and functional convergent evolution of snake venom proteinaceous toxins

**DOI:** 10.1186/s12915-021-01208-9

**Published:** 2022-01-07

**Authors:** Bing Xie, Daniel Dashevsky, Darin Rokyta, Parviz Ghezellou, Behzad Fathinia, Qiong Shi, Michael K. Richardson, Bryan G. Fry

**Affiliations:** 1 Institute of Biology Leiden, Leiden University, 2333BE, Leiden, The Netherlands; 2 Venom Evolution Lab, School of Biological Sciences, University of Queensland, St Lucia, 4072 Australia; 3 Australian National Insect Collection, Commonwealth Science and Industry Research Organization, ACT, Canberra, 2601 Australia; 4 Department of Biological Science, Florida State University, Tallahassee, FL 24105 USA; 5 Medicinal Plants and Drugs Research Institute, Shahid Beheshti University, Tehran, 1983969411 Iran; 6 Institute of Inorganic and Analytical Chemistry, Justus Liebig University Giessen, 35392, Giessen, Germany; 7 Department of Biology, Faculty of Science, Yasouj University, Yasouj, 75914 Iran; 8 Shenzhen Key Lab of Marine Genomics, Guangdong Provincial Key Lab of Molecular Breeding in Marine Economic Animals, BGI Academy of Marine Sciences, BGI Marine, BGI, Shenzhen, 518083 China; 9 BGI Education Center, University of Chinese Academy of Sciences, Shenzhen, 518083 China

**Keywords:** Snake venom, Evolution, Selection, 3FTx, Kunitz, Natriuretic, Lectin, P-III SVMP

## Abstract

**Background:**

The explosive radiation and diversification of the advanced snakes (superfamily Colubroidea) was associated with changes in all aspects of the shared venom system. Morphological changes included the partitioning of the mixed ancestral glands into two discrete glands devoted for production of venom or mucous respectively, as well as changes in the location, size and structural elements of the venom-delivering teeth. Evidence also exists for homology among venom gland toxins expressed across the advanced snakes. However, despite the evolutionary novelty of snake venoms, in-depth toxin molecular evolutionary history reconstructions have been mostly limited to those types present in only two front-fanged snake families, Elapidae and Viperidae. To have a broader understanding of toxins shared among extant snakes, here we first sequenced the transcriptomes of eight taxonomically diverse rear-fanged species and four key viperid species and analysed major toxin types shared across the advanced snakes.

**Results:**

Transcriptomes were constructed for the following families and species: Colubridae - *Helicops leopardinus*, *Heterodon nasicus*, *Rhabdophis subminiatus*; Homalopsidae – *Homalopsis buccata*; Lamprophiidae - *Malpolon monspessulanus*, *Psammophis schokari*, *Psammophis subtaeniatus*, *Rhamphiophis oxyrhynchus*; and Viperidae – *Bitis atropos*, *Pseudocerastes urarachnoides*, *Tropidolaeumus subannulatus*, *Vipera transcaucasiana*. These sequences were combined with those from available databases of other species in order to facilitate a robust reconstruction of the molecular evolutionary history of the key toxin classes present in the venom of the last common ancestor of the advanced snakes, and thus present across the full diversity of colubroid snake venoms. In addition to differential rates of evolution in toxin classes between the snake lineages, these analyses revealed multiple instances of previously unknown instances of structural and functional convergences. Structural convergences included: the evolution of new cysteines to form heteromeric complexes, such as within kunitz peptides (the beta-bungarotoxin trait evolving on at least two occasions) and within SVMP enzymes (the P-IIId trait evolving on at least three occasions); and the C-terminal tail evolving on two separate occasions within the C-type natriuretic peptides, to create structural and functional analogues of the ANP/BNP tailed condition. Also shown was that the *de novo* evolution of new post-translationally liberated toxin families within the natriuretic peptide gene propeptide region occurred on at least five occasions, with novel functions ranging from induction of hypotension to post-synaptic neurotoxicity. Functional convergences included the following: multiple occasions of SVMP neofunctionalised in procoagulant venoms into activators of the clotting factors prothrombin and Factor X; multiple instances in procoagulant venoms where kunitz peptides were neofunctionalised into inhibitors of the clot destroying enzyme plasmin, thereby prolonging the half-life of the clots formed by the clotting activating enzymatic toxins; and multiple occasions of kunitz peptides neofunctionalised into neurotoxins acting on presynaptic targets, including twice just within *Bungarus* venoms.

**Conclusions:**

We found novel convergences in both structural and functional evolution of snake toxins. These results provide a detailed roadmap for future work to elucidate predator–prey evolutionary arms races, ascertain differential clinical pathologies, as well as documenting rich biodiscovery resources for lead compounds in the drug design and discovery pipeline.

**Supplementary Information:**

The online version contains supplementary material available at 10.1186/s12915-021-01208-9.

## Background

Early snakes possessed mixed serous-mucous oral toxin glands inherited from the last common ancestor of Toxicoferan reptiles [1]. Extant snakes have evolved a number of different gland morphologies from this ancestral state [2, 3]. Some basal snakes such as *Cylindrophis* and *Eryx* have glands which are primarily serous (protein secreting) tissue which produce appreciable quantities of proteinaceous toxins (such as 3FTx with paralytic effects), with mucous secreting cells in the minority [3, 4]. In contrast, in the derived basal snake lineages which have secondarily evolved powerful constriction as a novel form of prey capture (boas and pythons), the glands were switched to a primarily mucous-secreting function in order to lubricate the large furred or feathered prey and facilitate their ingestion [3]. However, in these constricting snakes, trace levels of proteinaceous toxins (such as 3FTx and lectins) are still secreted as evolutionary relics [3]. These proteins are descended from ancestral toxins and can be detected by SDS-PAGE gel electrophoresis or via PCR amplification of the encoding genes and remain sufficiently similar to other snake toxins to produce false positives in antibody-based snake venom detection kits [3, 5].

The explosive radiation of the advanced snakes (superfamily Colubroidea [6]) was associated with the partitioning of the ancetral mixed glands into two discrete glands, one devoted to venom production, the other for mucous [2, 7]. The venom gland has subsequently evolved into an extraordinary diversity of morphological forms [2, 7, 8]. The venom systems of the lamprophiid lineage (including the genera *Atractaspis* and *Homoroselaps*), elapids, and viperids are homoplastic in that they have convergently evolved hollow fangs linked via tube-like ducts to the venom glands which are enclosed by powerful compressor muscles to increase the speed and efficiency of venom delivery. Similar compressor muscles have also evolved in at least three other lineages (*Brachyophis revoili*, *Dispholidus typus*, and *Gonionotophis capensis*) without the additional refinement of syringe-like venom-delivering dentition [2, 8].

Numerous morphological and developmental studies have ascertained that the venom-producing glands of all advanced snakes are homologous structures that develop from the primordium at the posterior end of the dental lamina [2, 7, 9–12]. Despite this demonstrated homology, the gland of rear-fanged snakes is often distinguished in the literature from that of front-fanged snakes through the use of the term ‘Duvernoy’s gland’. As noted previously [7], use of this term perpetuates a historical mistake that was made in the original designation of the gland by Taub in 1967 [13]. At that time, Taub agreed with earlier work that the post-orbital gland of rear-fanged snakes produced venom, citing studies from the early 1900s [4, 14], but considered the glands of viperids and elapids to be non-homologous to each other, and thus assumed the gland of the rear-fanged snakes were also not homologous to elapids or viperids. Crucially, the phrase ‘Duvernoy’s gland’ was not even suggested to highlight this erroneous non-homology but was simply suggested as a replacement for the name ‘parotid gland’ which was also occasionally assigned to this structure in rear-fanged snakes. It is now considered well-established that the venom glands of all colubroid snakes are homologous [2]. In fact, elapid and viperid glands are more closely related to the glands of rear-fanged snakes than they are to each other. Thus, the use of the term ‘venom gland’ for the homologous glands of all advanced snakes is the more appropriate than to refer to a paraphyletic array of morphologies as ‘Duvernoy’s glands’.

Just as the venom glands of advanced snakes are homologous, so are the venom-delivering teeth. Developmental uncoupling of the posterior sub-region of the tooth forming epithelium facilitated evolution of a wide range of highly modified posterior teeth, with tremendous diversity both in size and morphology [8, 15]. Enlarged rear-fangs—which have evolved on a myriad of occasions—and the three independent evolutions of hollow front fangs, all evolved from the same posterior teeth [12]. These teeth evolved to be farther forward in the mouth of the three front-fanged lineages due to shortening of the maxillary bone and the loss of more anterior teeth [12].

In a similar fashion to the homology of the morphological aspects of the venom system, modern evidence has accumulated for the homology venom gland toxins expressed across the advanced snakes. The discovery of three-finger toxins (3FTx) for the first time outside of elapid snakes [16] stimulated a phylogenetic analysis of all known toxin types, revealing the non-monophyly of a myriad of toxin types relative to the organismal relationships [17]. Several toxin families were found to be shared across all advanced snakes including 3FTx, acetylcholinesterase, C-type natriuretic peptides (CNP), kallikrein enzymes, hyaluronidase, kunitz, lectins, and snake venom metalloproteases (SVMP). As the species and their venom secretion and delivery system have diversified, so too have the venom proteins themselves. Accelerated evolution and rapid neofunctionalisation are common traits of snake venom gene families [18–23]. These genes are often subject to duplication that is sometimes followed by functional and structural diversification [24–27] and accelerated rates of sequence evolution [28, 29]. This diversification is possibly a result of selection for the ability to kill and digest different prey [30] or as part of a predator–prey arms race (e.g., [31, 32]).

Despite the evolutionary novelty of snake venom proteins, a comprehensive reconstruction of the molecular evolutionary history of major shared toxin types has not been undertaken. This has been in part due to the relatively low number of sequences available from rear-fanged species. Elapid and viperid snake species have been the focus of much more research because of their medical importance [33, 34]. To carry out these broad analyses, we obtained venom gland transcriptomes from eight rear-fanged species spanning the families Colubridae subfamilies Dipsadinae (*Helicops leopardinus*, *Heterodon nasicus*) and Natricinae (*Rhabdophis subminiatus*), Homalopsidae (*Homalopsis buccata*), and the Lamprophiidae subfamily Psammophiinae (*Malpolon monspessulanus*, *Psammophis schokari*, *Psammophis subtaeniatus*, and *Rhamphiophis oxyrhynchus*) as well as four viperid species spanning that family’s phylogenetic range (*Bitis atropos*, *Pseudocerastes urarachnoides*, *Tropidolaemus subannulatus*, and *Vipera transcaucasiana*). With these sequences, we were able to reconstruct the molecular evolutionary history of major shared toxin types and map their diversification patterns relative to the organismal relationships and functional changes in the venom. This allows us to evaluate the relative order of evolutionary events such as the diversification of Colubroidea, the partitioning of the venom glands into discrete mucous and protein-secreting units, diversifications in toxin families, and structural and functional novelties in toxin sequences. Since most Colubroid lineages possess the partitioned gland, partitioning is assumed to have occurred in the common ancestor of the superfamily and some researchers suspect that the specialization of this gland for venom production is one of the underlying causes for the explosive diversification of these snakes [2, 7, 8]. We know little about the toxins produced by these ancestral species, but phylogenetic analyses can offer suggestive evidence of when toxin duplication events may have occurred. Ancient duplication events should result in toxin phylogenies where toxins that are descended from one copy are more closely related to toxins descended from that same copy in distantly related snakes (orthologs) than they are to toxins descended from the other copy in the same organism (paralogs). In other words, an ancient gene duplication would lead to a toxin tree in which at least two toxin clades resemble the taxonomic tree. Conversely, more recent expansions of a given toxin family should lead to toxin clades that arose in, and are exclusive to, a specific colubroid lineage.

## Results

The annotation of the 10 assembled venom gland transcriptomes recovered 23 toxin families (Additional File 4). Of these, the most conspicuous evidence for extensive gene duplication and diversification was evident in 3FTx, CNP, CRiSP, kallikrein, lectin, and SVMP gene families and thus in-depth analysis of their molecular evolutionary history through sequence alignments, phylogenies, and signals of selection was undertaken. Signals of selection analyses were designed to identify genes, regions, or single amino acids diversifying under positive selection or constrained by negative selection by calculating the ratio of nonsynonymous nucleotide substitutions (dN) with that of synonymous substitutions (dS) (*ω* = dN/dS), which can be used as an indicator of selective pressure acting on a protein coding gene. *ω* > 1 implies positive selection (functional diversification), whereas *ω* < 1 implies purifying selection. Sites with *ω* values near 1 are thought to evolve neutrally. We performed a series of tests combining information from full sequence and site-based and in order to determine the most likely groups on which positive selection has been operating.

To examine the patterns of selection operating in different toxin families, we used an overall measure of *ω* for full-length coding sequences or particular regions as well as two independent site-specific analyses: Fast Unconstrained Bayesian App Roximation (FUBAR) which gauges the strength of consistent positive or negative selection and Mixed Effects Model of Evolution (MEME) which identifies individual sites that were subject to episodes of positive selection in the past [35–37]. The results of these site-specific tests can be visualised on the 3D protein structures to understand if particular regions of the protein are under different selection regimes.

### Three-finger toxins (3FTx)

The 3FTx family is one of the most abundantly secreted non-enzymatic components in several snake families, particularly colubrids and elapids [2, 16, 18, 21, 23, 38]. The ancestral activity of this toxin family is antagonistic binding to the *α*-1 subunit of the post-synaptic nicotinic acetylcholine receptor, resulting in flaccid paralysis [16, 38, 39]. The ancestral form is characterised by 10 cysteines, with the 2nd and 3rd cysteines lost in the elapid-specific forms [38]. The ancestral form has been referred to by various terms. One is the erroneous ‘weak toxin’ designation which was coined due to their perceived low levels of toxicity. This perception was due to the testing upon mammalian targets, but these models were evolutionarily misleading since the majority of the snakes that secrete the ancestral type are rear-fanged species which specialise on diapsids such as birds and lizards. Such poor model choice even led to some rear-fanged species, such as *Boiga irregularis*, being erroneously referred to as non-venomous [40, 41]. Subsequent testing on bird and lizard assay systems revealed these venoms to have extremely taxon-selective potent toxicity [16, 42–45]. Another term often used for the basal-type 3FTx to distinguish them from the elapid-specific derived form that lacks the 2nd and 3rd cysteines, is the ‘non-conventional’ designation [46]. As 'conventional' and 'non-conventional' are evolutionarily meaningless terms, we prefer to use the term ‘basal’ since this captures the evolutionary history they share with the myriad of derived forms present in elapid venoms.

**Fig. 1:**
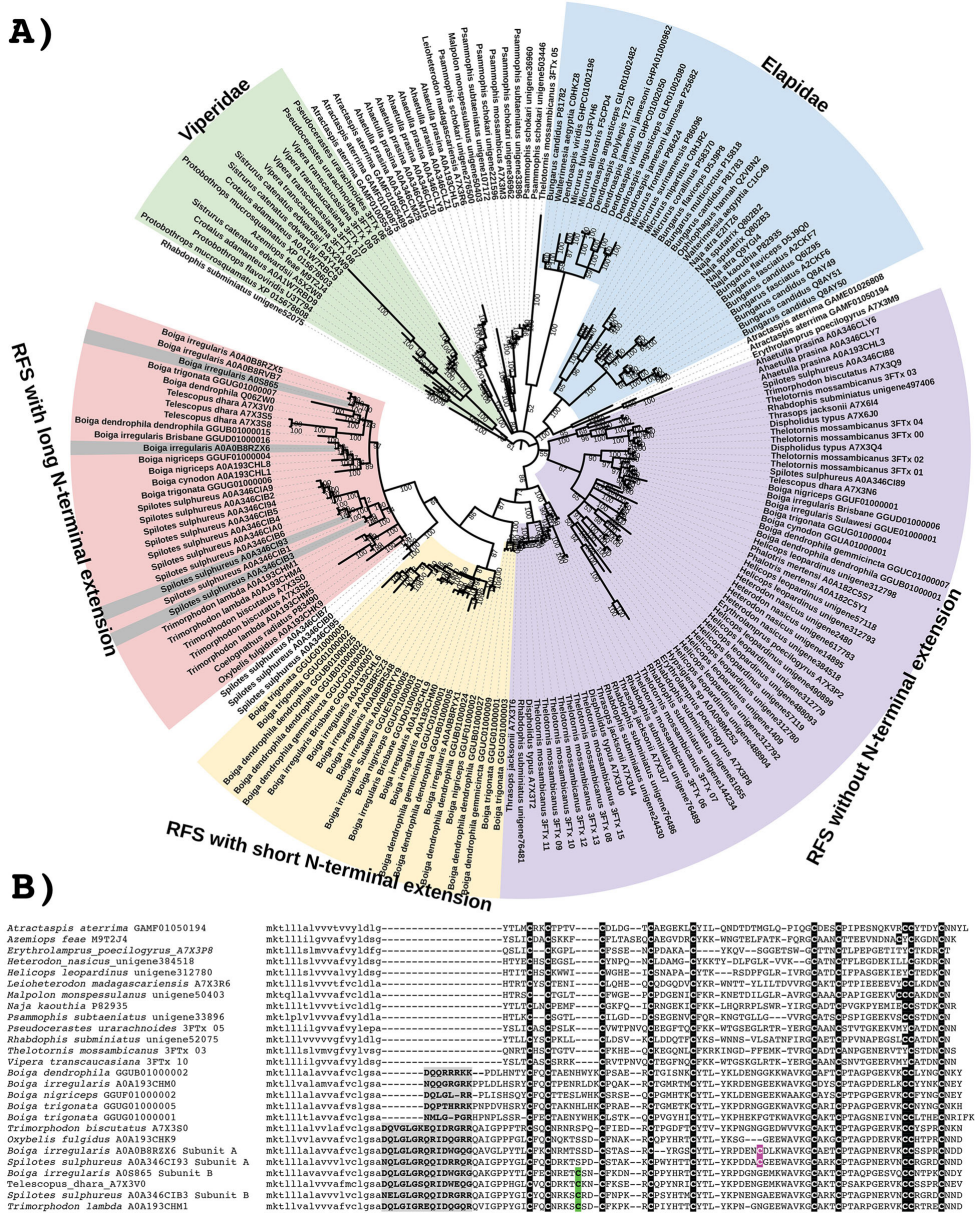
**A** Molecular phylogenetic reconstruction of 3FTx showing extensive duplication preceding organismal diversification combined with lineage-specific duplication events. Grey shaded sequences represent the dimeric forms that have evolved convergently in *Boiga* and *Spilotes*. Additional Files contain sequence alignment used for constructing phylogenetic tree (Additional file 1), tree output file (Additional file 2), and zoomable high-resolution tree image (Additional file 3). RFS = rear-fanged snake. **B** Sequence alignment of representative basal-type 3FTx. Ancestral cysteines are shaded in black. Newly derived dimeric inter-chain cysteines shaded in colour. Propeptide domain shaded in grey. Signal peptide amino acids shown in lowercase

In some colubrids, a novel form of basal 3FTx includes an insertion subsequent to the signal peptide that creates a propeptide region and also an extension of N-terminus the processed peptide (Fig. 1). There are two subtypes of these extended toxins which both produce processed peptides of similar length, but they differ in two important aspects. First is the length of the propeptide region, which is 6–8 amino acids in the form restricted to the *Boiga* genus, and 14 amino acids in the form widespread across colubrid venoms. Whether the post-translationally cleaved propeptide region is secreted and plays a role in the envenomation process—thus representing a new toxin class (as described for CNP and SVMP below)—is a rich area for future research. A second notable difference is that the longer form cleaves before a glutamic acid. The first study to isolate a 3FTx from a non-elapid venom revealed that this cleavage results in a pyroglutamic acid which unless enzymatically cleaved impedes Edman degradation sequencing [16]. The ancestral form of the 3FTx gene has three exons, and the N-terminal extension of the processed peptide and the propeptide domain are coded by a newly inserted exon [47]. Thus it is also notable that the forms with this additional exon also have inserted residues between the second and third ancestral cysteine (Fig. 1). It is also within the form with the longer extension that the additional cysteine has evolved that forms one half of the inter-chain disulphide bond of unique dimeric forms convergently evolved in *Boiga/Telescopus* and *Spilotes/Trimorphodon* (as described below) [21, 42, 43, 45].

The functional implications of the N-terminal extension of the processed peptide have not been elucidated, but the extensive duplication of both extension forms suggests that they have been fixed and amplified under positive selection pressure. It is notable that the two forms (short and long extension) do not form a monophyletic group (red and yellow clades in Fig. 1), with non-extended sequences from *Spilotes* intervening. This suggests two competing hypotheses: (1) short and long extensions arose convergently, or (2) the *Spilotes* sequences represent the secondary loss of a singl-evolved extension. In light of the stark differences in the amino acids making up the short versus long extension forms (Fig. 1B), a convergent evolution of the N-terminus extension seems more likely, which in turn indicates a strong selective pressure in favour of this trait, either in function of potency or selectivity.

With high posterior support values, our phylogenetic analyses confirm previous results [45] which suggested that the dimeric form of 3FTx evolved on multiple occasions, with the *Boiga* A and B subunits more closely related to each other than they are to either the *Spilotes* A or B subunits and vice versa (Fig. 1). If the complexes had only evolved once we would expect the A subunits from both species to be more closely related to each other than the B subunits. As the inter-chain cysteines are located in the same positions for both, this suggests structural constraints acting on the evolution of the inter-chain disulphide bonds whereby there are extreme limitations in geographical positions amendable to dimer formation. In addition, for both the *Boiga* and *Spilotes* forms, the B subunit is present in other genera from the same geographic region and taxonomical subclade, *Telescopus* for *Boiga* and *Trimorphodon* for *Spilotes* (Fig. 1). This suggests that the B subunit evolved in the last common ancestor of *Boiga* and *Telescopus* and again convergently in the last common ancestor of *Spilotes* and *Trimorphodon*. The timing of convergent evolution of the A subunit in each case remains to be elucidated as while only *Boiga* or *Spilotes* sequences are available, in-depth sequencing of related genera has not been undertaken. Absence of evidence therefore cannot be considered as evidence of absence. Thus, while the current data suggests that the B subunit evolved earlier in each case and that the convergent origins of an A subunit happened more recently in *Boiga* and *Spilotes*, this perception may change as more sequencing of more species is undertaken. However, if the B-chain evolved before the A-chain, the free cysteines present in the B-chains may allow the formation of B-chain homodimeric toxins in the lineages that lack the A-chains.

**Fig. 2:**
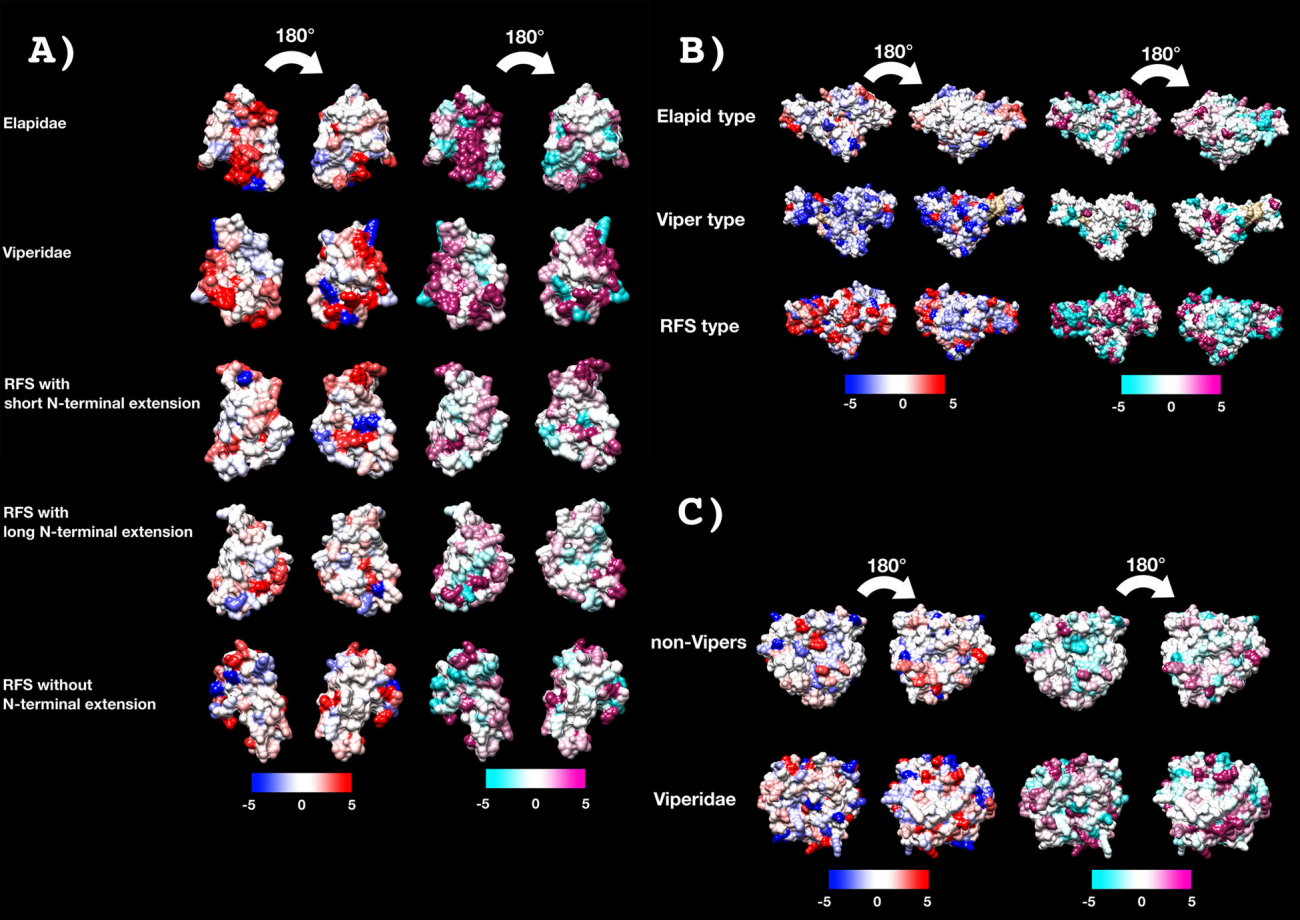
Molecular modelling of showing sites under selection by FUBAR (left) and MEME (right) colour coded to show sites that are negatively (blue and turquoise, resp), neutrally (white), or positively (red and purple resp.) selected. Protein models show front and back views coloured according to FUBAR’s estimated strength of selection (*β*-*α*, left) and MEME’s significance levels (right). See Table 1 for values and Additional file 4 for modelling templates. **A** 3FTx, **B** CRiSP, **C** kallikrein

The rates of evolution calculations showed all basal 3FTx forms to be evolving under positive selection and with sites under selection (Fig. 2A and Table 1). It should be noted that the apparent higher rate of overall evolution of the viper sequences may be skewed due to only a few sequences being available for this 60-million-year-old clade. Thus, as more sequences become available, it is hypothesised that the *ω* value will be significantly reduced.

**Table 1:** Molecular evolutionary rates

Clade	***ω***	FUBAR (−)^**a**^	FUBAR (+)^**b**^	MEME^**c**^	FUBAR and MEME^**d**^
**3FTx** (See Fig. 2A for modelling)					
**Elapidae**	1.17	5	13	15	10
Rear-fanged snakes with short N-terminal extension*	1.51	2	8	12	7
Rear-fanged snakes with long N-terminal extension*	1.37	2	14	22	14
**Rear-fanged snakes without N-terminal extension**	1.30	8	20	27	19
**Viperidae**	2.38	2	15	15	10
**CRiSP** (See Fig. 2B for modelling)					
**Elapidae-dominated clade**	1.34	9	33	29	22
**Viperidae clade**	1.31	15	37	63	32
**Rear-fanged snake dominated clade**	1.25	36	35	52	32
**Kallikrein** (See Fig. 2C for modelling)					
**Non-viperids**	0.70	26	11	11	6
**Viperidae**	1.01	73	73	100	67
**Kunitz** (See Fig. 9A for modelling)					
**Viperidae clade**	0.96	1	8	8	4
**Non-viperid clade**	1.18	5	10	13	6
**Neurotoxins** **Dendrotoxins**	2.10	0	6	7	3
**Neurotoxins** **Bungarotoxins**	1.23	15	25	30	22
**Plasmin inhibitors** ***Daboia***	0.80	1	0	2	0
**Plasmin inhibitors** ***Oxyuranus/ Pseudonaja***	0.62	2	0	0	0
**Lectin** (See Fig. 9B for modelling)					
**Ancestral (monomeric)**	0.72	44	14	31	14
**Derived (dimeric)** **Non-viperids excluding** ***Helicops*)**	0.97	6	16	21	10
**Derived (dimeric)** ***Helicops***	1.16	0	6	7	3
**Derived (dimeric)** **Viperidae-alpha**	1.40	23	54	60	49
**Derived (dimeric)** **Viperidae-beta**	1.27	17	45	54	38

^a^Number of codons under negative selection according to FUBAR

^b^Number of codons under positive selection according to FUBAR

^c^Number of codons under episodic diversifying selection according to MEME

^d^Number of codons that fit criteria ^b^ and ^c^

*Propeptide region not included in rate of evolution calculations

### C-type natriuretic peptides (CNP)

**Fig. 3:**
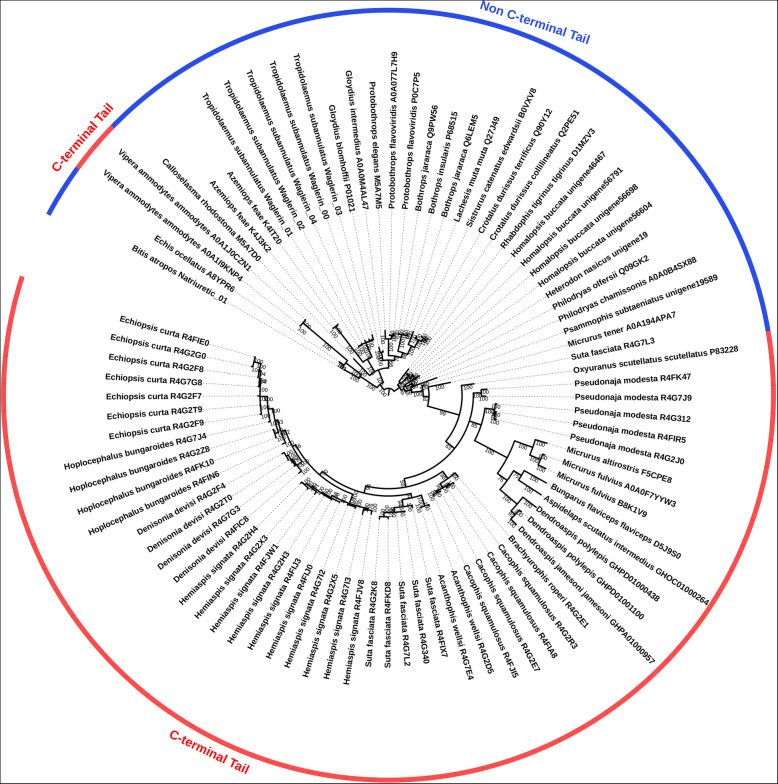
Molecular phylogenetic reconstruction of the C-type natriuretic peptide gene showing the convergent evolution of C-terminal tail not present in the ancestral toxin, and the subsequent elapid-specific explosive duplication and diversification of this derived trait. Additional files contain sequence alignment used for constructing phylogenetic tree (Additional file 5), tree output file (Additional file 6), and zoomable high-resolution tree image (Additional file 7)

**Fig. 4:**
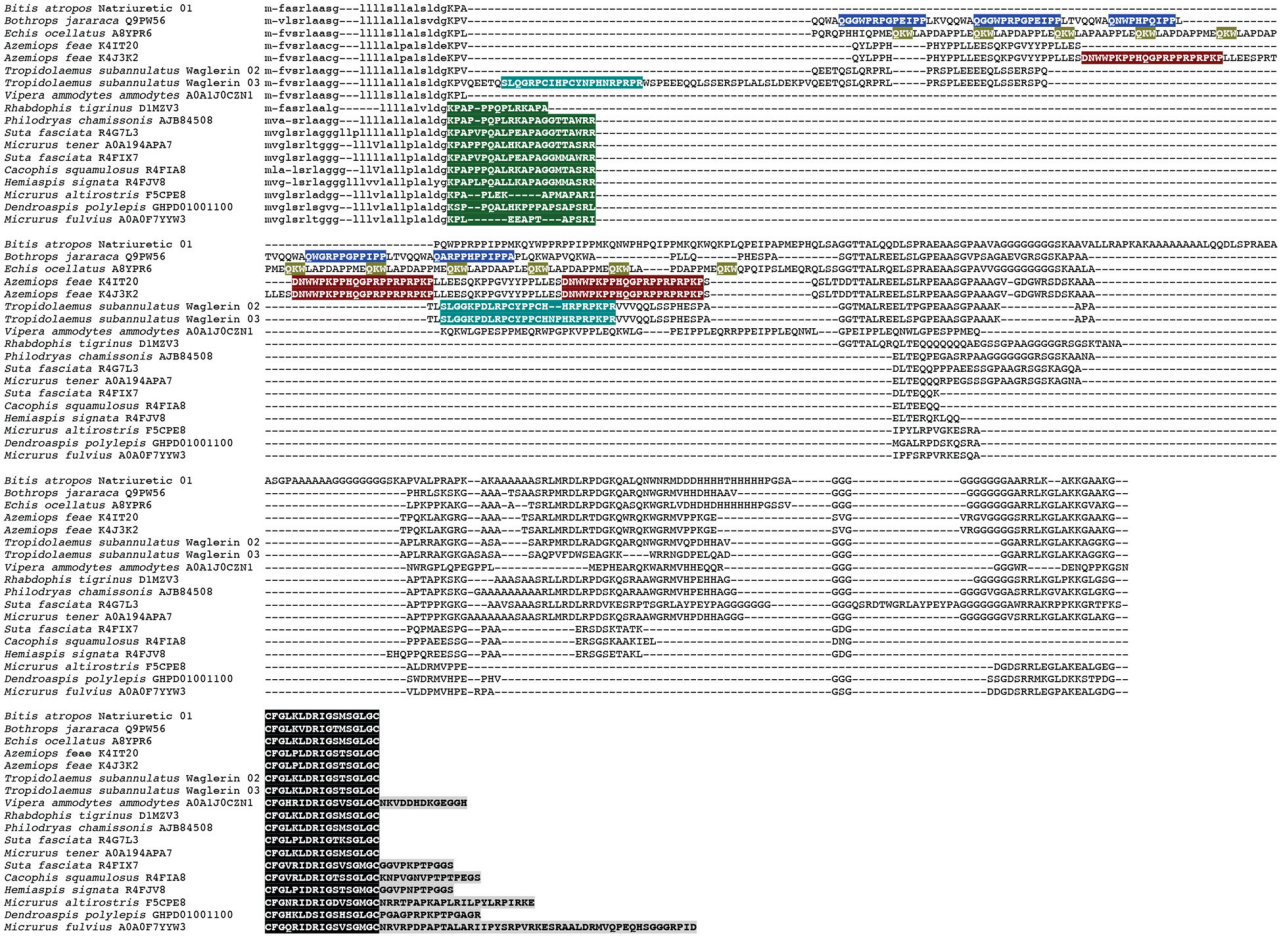
Sequence alignment of representative C-type natriuretic precursors. Signal peptide shown in lowercase. Newly evolved, post-translationally liberated peptides shown in coloured highlights, with the basal natriuretic peptide in black with the derived C-terminal tails shown in grey highlights

Natriuretic peptide toxins potently induce hypotension by binding to receptors located on the endothelium, thereby relaxing aortic smooth muscle [48]. Snake venom natriuretic peptides were initially thought to originate from two different recruitment events: an elapid venom form with a C-terminal tail similar to the endogenous ANP or BNP type, and a viper venom form lacking a C-terminal tail related to the CNP type [17]. It was not until full-length gene sequences were obtained that subsequent molecular phylogenetic reconstructions revealed that all snake venom natriuretic peptides originated from the tailless CNP type [49, 50]. Molecular phylogenetic analysis in the curent study showed that the derived tailed form has evolved on two occasions, once in the elapids and once in the viperids, with viperid venoms dominated by the basal tailless form while elapid venoms are dominated by the derived tailed form. And consistent with elapids and viperides independently evolving tailed forms, all rear-fanged species possess the tailless form (Figs. 3 and 4). The evolution of the C-terminal tail switches the binding specificity from natriuretic peptide receptor type GC-B (CNP receptor) to type GC-A (ANP/BNP receptor) [51–53]. The evolutionary advantage of targeting the respective endogenous receptor is not known and consequently is a rich area for future research. The evolution of the tailed form in elapids occurred at the base of the family, and some elapids (e.g. *Suta* and *Micrurus*) express both the tailed and tailless forms. Subsequent to the evolution of the tailed form in the elapids, there was a secondary loss of the poly-G motif in the precursor region just upstream of the natriuretic peptide domain. The timing of the evolution of the viperid tailed form is enigmatic. High-quality, full-length mRNA precursors of tailed forms from viperides are only known from *Vipera* [54, 55]. But highly similar tailed versions of the post-translationally liberated natriuretic peptide domain have been reported from the crude venoms of *Macrovipera* and *Pseudocerastes* [56, 57]. In addition, the mRNA precursor for a tailed version has been reported for *Cerastes* (Genbank A8YPR9|SVMI1), but as the ring motif contains amino acid deletions not seen in any other natriuretic peptide, the sequence quality must be regarded as provisional. Regardless, as this genus is phylogenetically closely related to *Macrovipera*, *Pseudocerastes*, and *Vipera*, the presence of the C-termnal tail appear authentic. Thus, in consideration of the organismal phylogeny of viperid snakes [58], the evolution of the C-terminal tail appears to have evolved in at least the last common ancestor of the clade consisting of *Cerastes*, *Daboia*, *Echis*, *Macrovipera*, *Montivipera*, *Pseudocerastes*, and *Vipera.* As this clade has not been the subject of intensive transcriptome sequencing, it is anticipated that as additional transcriptomes are sequenced, more tailed forms will be recovered. The form recovered from the more distantly related *Bitis* in this study was the basal tailless form. In contrast, the pit viper venom gland transcriptomes have been extremely deeply sequenced and only tailless form mRNA precursors have been recovered. Curiously, tailed forms have been reported as present in the venoms of *Crotalus durissus cascavella* and *Crotalus oreganus abyssus* [59–61]. In the light of these two species being distant to each other within this genus, this suggests that tailed forms should be widespread. However, this genus has been the subject of intense transcriptome studies, and no single one has recovered the tailed form. The *Crotalus* tailed forms were reported using Edman degradation sequencing, an old technique which was notoriously error prone. Thus, laboratory error cannot be ruled out. Therefore, until these two subspecies can be the subject of in-depth primer-directed sequencing, the presence of tailed forms in pit viper venoms must be viewed with caution. If confirmed, however, phylogenetic analyses using full-length precursor sequences will provide the crucial insights as to whether tailed forms evolved once or multiple occasions within viperids.

In addition to coding for the natriuretic peptide, the propeptide region of the genes has been hyper-mutated to code for a diverse range of post-translationally cleaved peptides that exert additional pathophysiological actions (Fig. 4). A similar scenario arose independently in Anguimorpha lizard venoms, where the natriuretic peptide gene was recruited for use as a toxin and the propeptide region has also become hyper-mutated in anguid and helodermatid lizards to encode for additional toxins that are post-translationally cleaved [62]. In the case of the snakes, the additional toxins encoded in the propeptide region range from hypotensive bradykinin-potentiating peptides [63] to neurotoxic (azemiopsins from *Azemiopis* ssp. [64, 65] and waglerins from *Tropidolaemus* sp [66–68]). A series of proline-rich peptides were isolated from the venom of *Dendroaspis angusticeps* as a novel toxin family, but the precursor genes could not be identified [69]. However, an *Oxyuranus* precursor (UniProt ID: P83228) was available at the time which showed significant similarity to this peptide in an early stretch of the propeptide domain. Comparison to sequences obtained in this study and obtained from public databases showed that this novel toxin type evolved within the propeptide domain subsequent to the divergence by viperids, but is ubiquitous in rear-fanged snakes and elapid precursors (Fig. 4). As they remain pharmacologically uncharacterised, their role in envenomation has yet to be determined. Consequently, they represent a promising area for future research. An important caveat is that the study which first described them documented post-translational modifications (glycosylation) in the peptides [69]. Synthetic forms without these modifications may have skewed or obviated bioactivities, so any material tested must either be native forms isolated from the venom, or recombinantly produced in a vertebrate cell expression system to ensure proper glycosylation occurs. Such post-translational modifications have been shown in other toxin classes to be essential for bioactivity, for example the cholecystoxin from *Varanus varius* venom which a sulfotyrosine was essential for activity, while the form with a normal (non-sulfated) tyrosine was completely inactive [70].

The hypervariability of the propeptide domain stands in stark contrast to the extreme conservation of the natriutetic peptide encoding domain (Fig. 4). The variability within the propeptide domain was so great as to make rates of evolution calculations impossible due to the extremely diffuse alignment within this region. The natriuretic peptide domain, however, was under strong negative selection in elapids (*ω* of only 0.06, with no sites under positive selection) and rear-fanged snakes (*ω* of only 0.07, with no sites under selection). While the *ω* value was higher in viperids (0.45), it still displayed significant negative selection pressure, and again there were no sites under positive selection. Such an extraordinary contrast reflects the high level of conservation of the endogenous targets as the natriuretic peptide receptors themselves are extremely conserved among vertebrate lineages (e.g. uniprot accession code P18910 (*Rattus norvegicus*) and Genbank accession code XM_042784559 (*Tyto alba*). Thus, the extreme conservation of the target imparts a correspondingly extreme negative selection upon the toxin in order to preserve the pathophysiological hypotensive action. On the other hand, the propeptide domain has the evolutionary freedom necessary for functional exploration and the emergence of novel toxin types. A similar pattern has been previously reported in amphibians and may be a widespread evolutionary mechanism for the origin of new peptides and bioactive functions in the precursor proteins of existing toxins [71].

### Cysteine-rich secretory proteins (CRiSP)

**Fig. 5:**
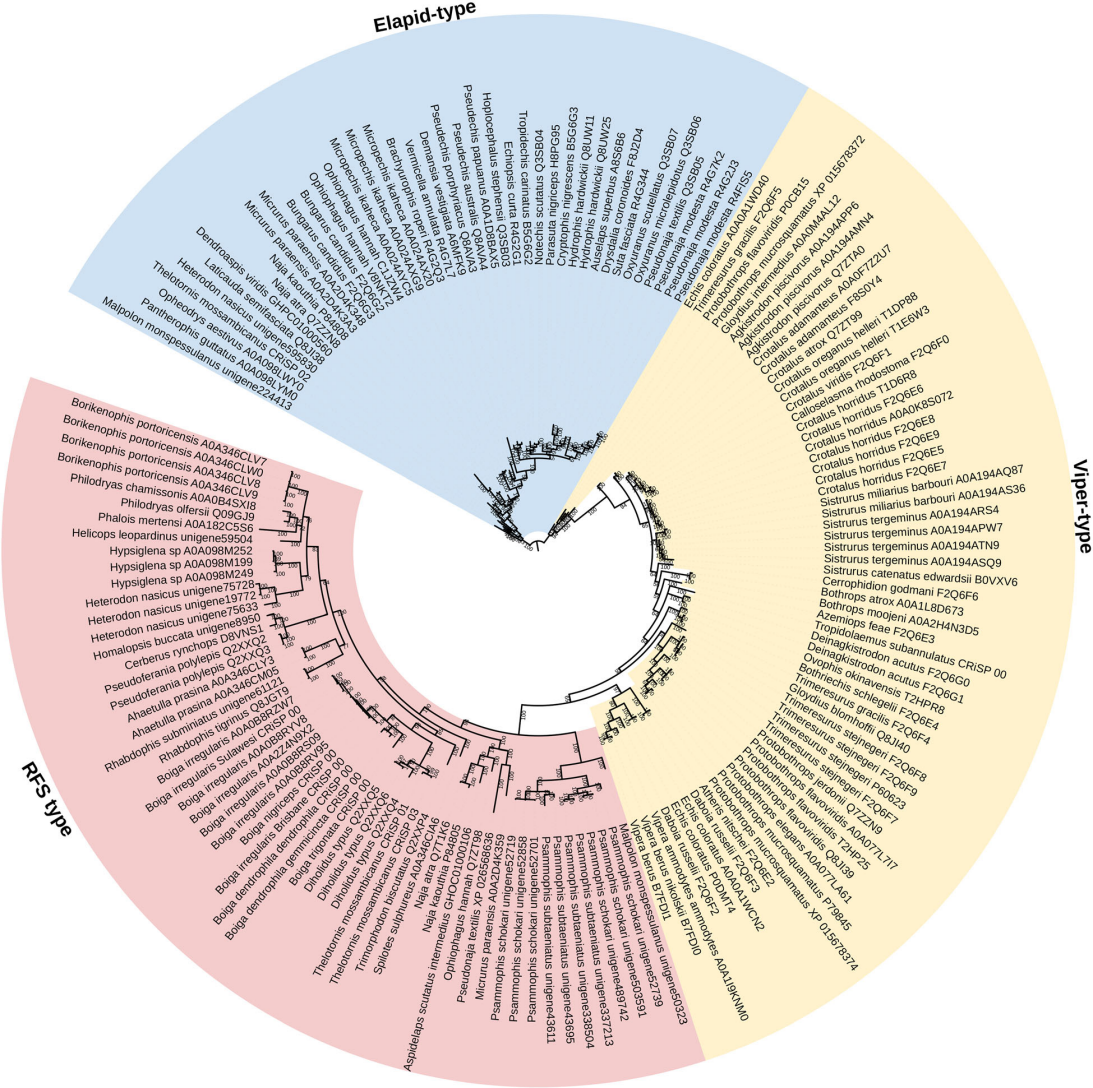
Molecular phylogenetic reconstruction of the CRiSP toxins showing the lineage-specific amplification of particular form. RFS = rear-fanged snake. While both the RFS and elapid-type contain other lineages, they are in extremely limited amounts and thus the nomenclature used reflects the dominant lineage expressing a particular form. See Table 1 for values. Additional Files contain sequence alignment used for constructing phylogenetic tree (Additional File 8), tree output file (Additional File 9), and zoomable high-resolution tree image (Additional File 10)

Phylogenetic analysis supports the classification of three distinct CRiSP types, with two characterised as Elapidae and Viperidae types respectively, while a third is restricted to rear-fanged snakes (Fig. 5). The three types represent well-delineated partitions in the tree, indicating a structural change at the origins of Elapidae and Viperidae, and the three clades showed moderate positive selection with *ω* values ranging 1.25 to 1.34, but presented appreciable selection sites, with the viperid and rear-fanged clades showing significantly more (32 each) than the elapids (22) (Fig. 2B and Table 1). The pathophysiological actions of CRiSP proteins are poorly defined and as a result the selection pressures leading to distinct forms being amplified and the different sites under selection between the three clades remain unclear. This is in spite of CRiSPs being major components in many rear-fanged snake venoms [2, 18, 72, 73]. Of the few that have been experimentally characterised, ion channel-binding neurotoxic effects are the most commonly reported activity [74–83]. Because the role in prey capture for CRiSP proteins remains uncertain, and they have extensively diversified independently within the different lineages and are subject to positive selection pressure, they represent a rich area for future structure-function studies.

### Kallikrein enzymes

**Fig. 6:**
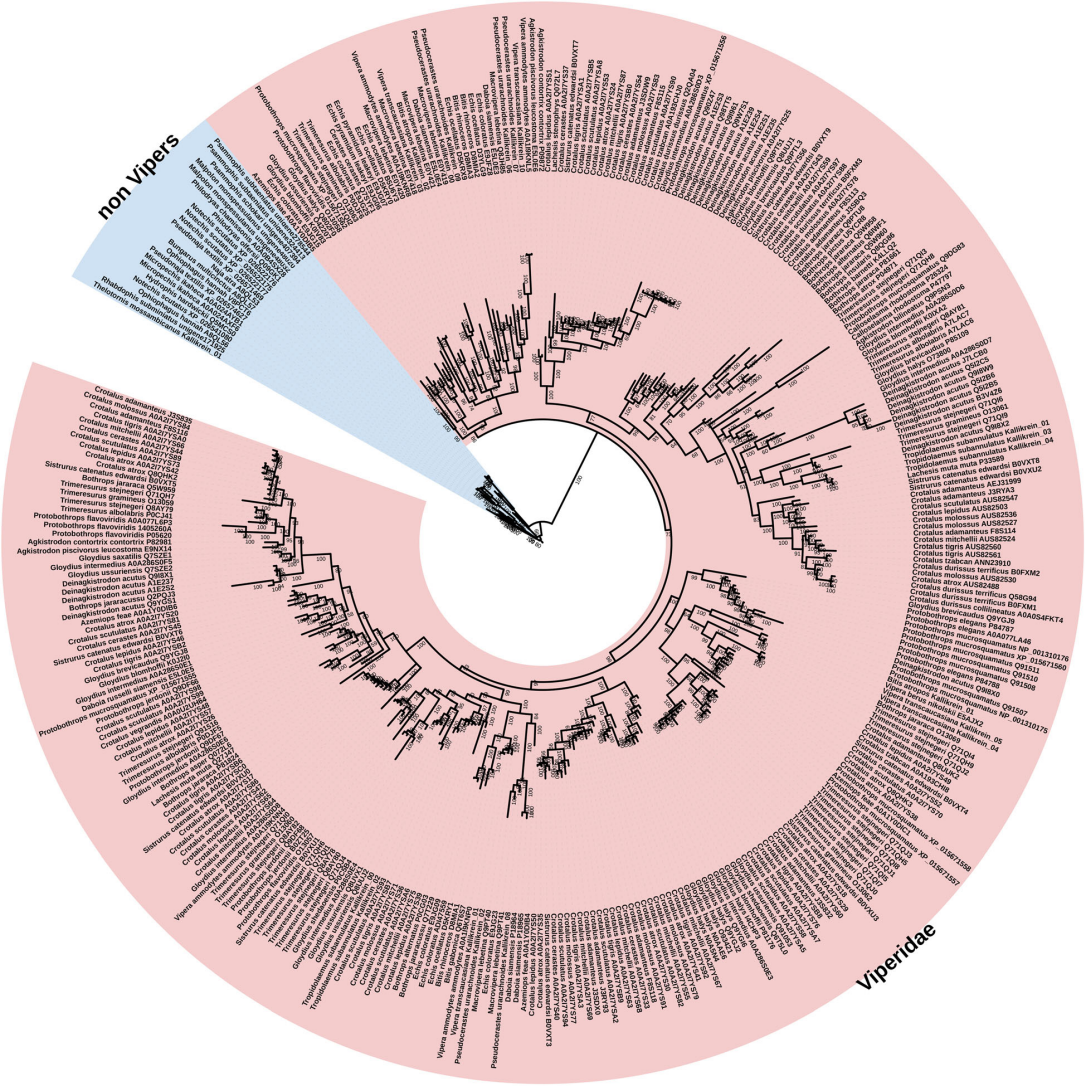
Molecular phylogenetic reconstruction of kallikrein toxins showing the lineage-specific explosive duplication and diversification in viperids. Additional Files contain sequence alignment used for constructing phylogenetic tree (Additional file 11), tree output file (Additional file 12), and zoomable high-resolution tree image (Additional file 13)

Kallikrein-type serine proteases are one of the most highly expressed toxin types in viperid snake venoms but are expressed at dramatically lower levels in other lineages. The relative evolutionary rates are reflective of this, with the viperid sequences displaying over 10 times the number of sites under selection (Fig. 2C and Table 1). Phylogenetically, the non-viperid snakes cluster together, indicating that the explosive radiation of kallirekins in viperid snakes occurred subsequent to divergence of this lineage from the other colubroid snakes (Fig. 6). Consistent with the dramatic structural and functional diversification within the viperids, the phylogenetic analysis revealed evidence of extensive gene duplication in the last common ancestor of the viperid snakes and significant variations in sites under selection. In contrast, for the colubrid and elapid snakes, the sequences follow organismal relationships. Within the viperid snakes, a wide range of toxic activities have been characterised, ranging from coagulotoxic (ranging from procoagulant activation of coagulation Factors FV, FX, and prothrombin, and anticoagulant depletion of fibrinogen levels, and anticoagulant activation of coagulation factors Protein C and plasminogen), to hypotension inducing cleavage of kininogen to release kinins [84]. The bioactivity of non-viperid forms remains in its infancy, but destructive cleavage of fibrinogen has been documented [85, 86]. These results suggest that fibrinogenolysis was an ancestral activity of the toxin family in advanced snakes and are also consistent with the hypothesis that the snake toxins are homologous with those highly expressed in the venoms of Angumorpha lizards, which also potently degrade fibrinogen [70, 87, 88].

### Kunitz peptides

**Fig. 7:**
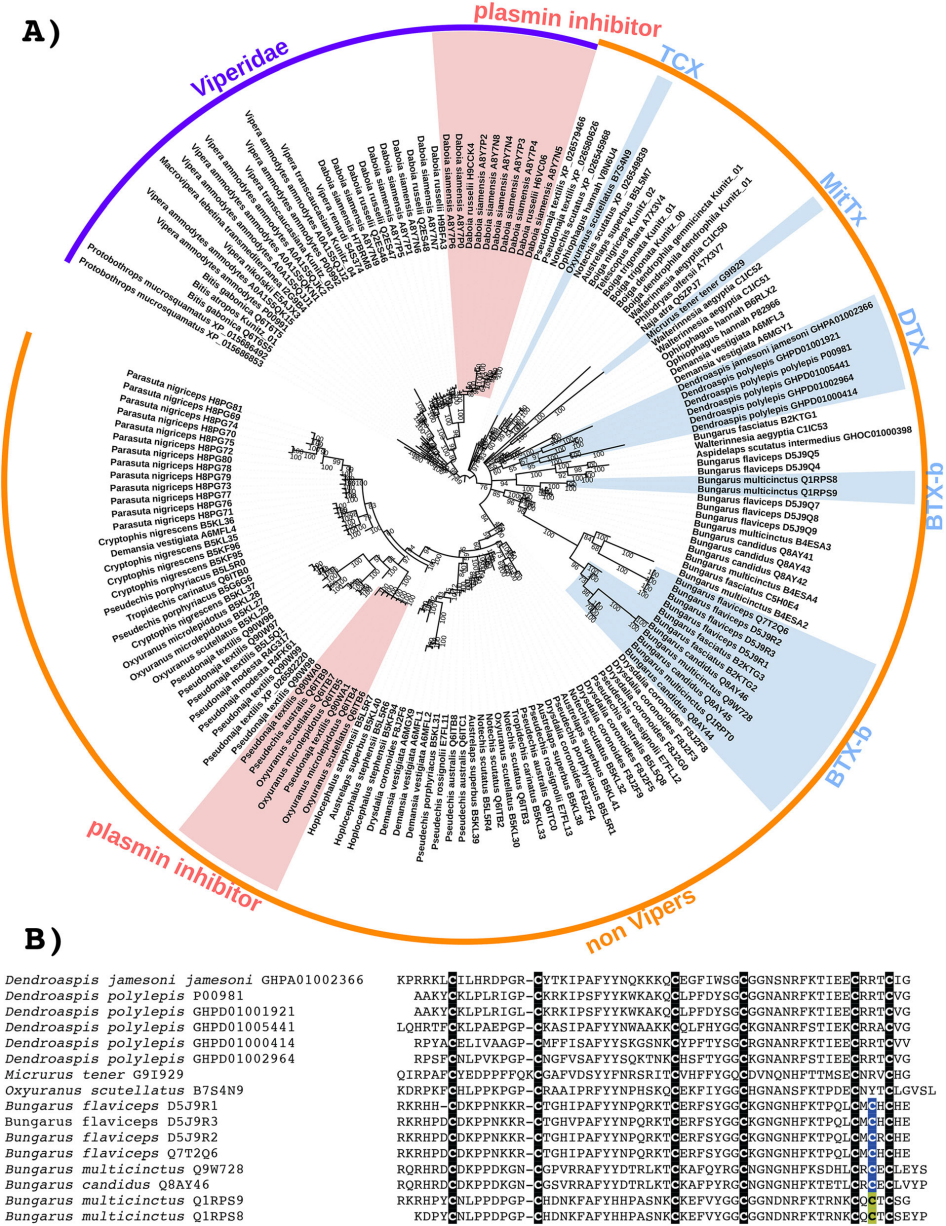
**A** Molecular phylogenetic reconstruction of kunitz toxins. The convergent evolution of coagulotoxins is shaded in red, while the convergent evolution of neurotoxins is highlighted in blue. TCX = taicatoxin. DTX = Dendrotoxin. BTX-b = β-bungarotoxins (characterised by the presence of a novel cysteine as per Fig. 7B). Additional Files contain sequence alignment used for constructing phylogenetic tree (Additional file 14), tree output file (Additional file 15), and zoomable high-resolution tree image (Additional file 16). **B** Sequence alignment of representative derived neurotoxic forms of kunitz peptides. Shown are *Dendroaspis* K_V_1.1 voltage-gated potassium channel blocking dendrotoxins; *Micrurus* acid-sensing ion channel ASIC1 activating MitTx; *Oxyuranus* cardiac voltage-dependent L-type calcium channels (Ca_V_) blocking taicatoxin; and *Bungarus* voltage-gated potassium channels (*K*_V_) blocking bungarotoxins. The convergent evolution of inter-chain cysteines in the *Bungarus* sequences are indicated by different highlight colours

Despite their high expression levels in many venoms, kunitz peptides have been the subject of relatively little research. Most testing has indeed focused on defining them as ‘chymotrypsin’ or ‘trypsin’ inhibitors [89], which are unlikely to be evolutionarily significant targets for venom toxins. Those that have been functionally characterised for biologically relevant activities tend to display coagulotoxic or neurotoxic effects [89]. Our analyses indicate that these toxins are extremely phylogenetically diverse and may well possess further novel functions (Fig. 7).

Diverse kunitz peptides have been characterised as neurotoxins, and our phylogenetic analysis combined with differences in sequence, structure, and function suggest that the evolution of this derived activity has occurred on four independent occasions (Fig. 7). These include monomeric toxins and members of multimeric toxin complexes. Dendrotoxins are monomeric toxins from *Dendroaspis* venoms that selectively block K_V_1.1 voltage-gated potassium channels [90]. Kunitz peptides that are subunits of multimeric neurotoxins may be associated through non-covalent interactions (MitTx and taicatoxins) or covalently linked (beta-bungarotoxins). Intriguingly, all such multimers are heteromeric and include PLA_2_ toxins as subunits. MitTx is a complex of one kunitz subunit and two PLA_2_ subunits that activates acid-sensing ion channel ASIC1 to cause intense pain as part of the defensive arsenal of *Micrurus tener* [91, 92]. Taicatoxin was discovered in the venom of *Oxyuranus scutellatus* and is a complex toxin consisting of one 3FTx subunit, one PLA_2_ subunit, and 4 kunitz subunits that blocks cardiac voltage-dependent L-type calcium channels (Ca_V_) [93]. β-bungarotoxins from *Bungarus* venoms are voltage-gated potassium channels (*K*_V_) blocking heterodimers consisting of a kunitz peptide disulphide-linked to a PLA_2_ subunit via a newly evolved cysteine not found in other kunitz peptides, linked to a matching novel cysteine in the PLA_2_ subunit that is also not found in non-bungarotoxin PLA_2_ toxins. Our phylogenetic analysis indicates that the characteristic cysteine in β-bungarotoxin kunitz peptides evolved independently on two different occasions (Fig. 7). In each case, the cysteine is in the same position, suggesting strong selection pressures due to inter-chain structural constraints. However, consistent with the phylogenetic placement into two distinct clades, each type differs in the flanking amino acids on either side of the cysteines. Intriguingly, there appears to have been a secondary loss of this trait occurring in the one of the kunitz peptide clades β-bungarotoxins, with the sequences C5H0E4 and B4ESA2 lacking the diagnostic and structurally necessary cysteine despite being nested within a clade of kunitz peptides containing this cysteine (Fig. 7).

**Fig. 8:**
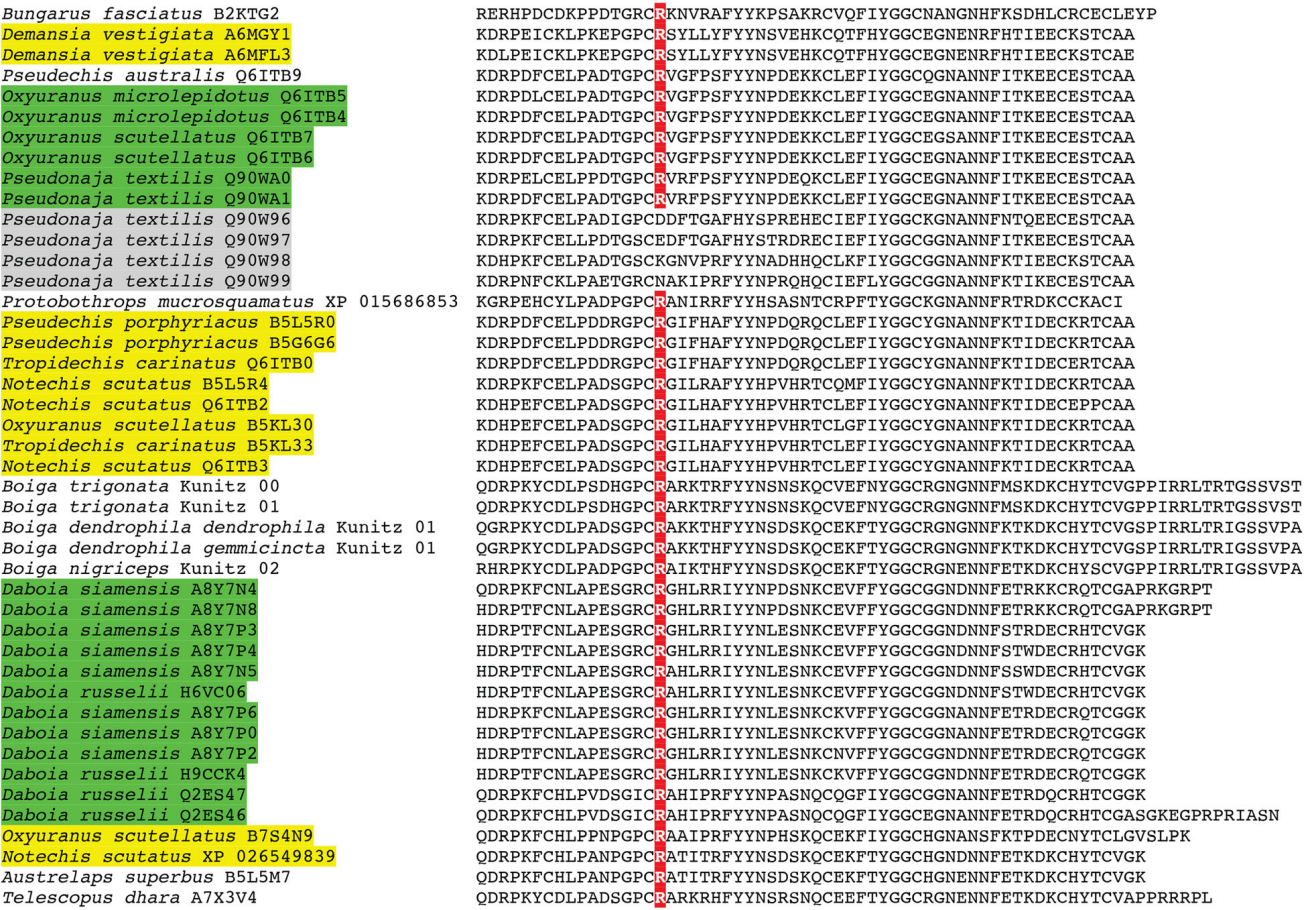
Sequence alignment of representative plasmin-inhibiting derived forms of kunitz toxins, with the functionally important arginine residue shaded in red. Procoagulant species with confirmed plasmin-inhibition are shaded in green. Procoagulant species which contain derived kunitz forms with the functionally important arginine but with phylogenetically distinct sequences that have not been functionally confirmed as plasmin-inhibiting are highlighted in yellow. *Pseudonaja* variants which lack the diagnostic arginine and have been tested for bioactivity and confirm the lack of plasmin inhibition activity are highlighted in grey

Other derived kunitz peptides have the pathophysiological action of inhibiting the clotting regulatory enzyme plasmin, which breaks down blood clots in the body. Unsurprisingly, plasmin inhibitors have been isolated and characterised from venoms which are powerfully procoagulant through the activation of Factor Xor prothrombin. The venoms allow the snakes to subjugate their prey by triggering the rampant production of endogenous thrombin, leading to the formation of enough blood clots to induce debilitating and lethal strokes [94, 95]. Such toxins have been well-described for the *Daboia* genus within the Viperidae family, and the *Oxyuranus/Pseudonaja* clade within the Elapidae family (Figs. 7 and 8). While *Daboia* venoms produce procoagulant toxicity through the activation of Factor X and *Oxyuranus* and *Pseudonaja* through the activation of prothrombin, both converge on the same functional outcome: the production of high levels of endogenous thrombin which convert fibrinogen to fibrin. Phylogenetic analysis (Fig. 7) reveals that they show convergent neofunctionalisation of the kunitz peptide such that they inhibit plasmin, thereby prolonging the half-life of the blood clots formed by the venom. Both species also show convergent modification of the same key residue into an arginine, which has been shown to be critical for activity. Structure-function studies (consisting of replacing the key arginine) combined with testing of natural variants lacking this arginine, underscore the critical importance of having an arginine at this position in order to inhibit plasmin, with variants (mutant or natural) lacking this arginine not being able to inhibit plasmin [96, 97]. Intriguingly, other phylogenetically distinct sequences contain this arginine (Fig. 8), the majority of which are in species with procoagulant venoms. It is also notable that there are additional *Oxyuranus* variants that are phylogenetically distinct from the functionally characterised plasmin inhibitors from *Oxyuranus* and *Pseudonaja* venoms, suggesting that *Oxyuranus* may have evolved plasmin-inhibiting kunitz peptides on multiple occasions. While the presence of the key functional arginine in the diagnostic position is strongly suggestive of the ability to inhibit plasmin, and thus contribute to the net procoagulant toxicity, functional studies are needed to confirm that these various other arginine-containing peptides are indeed plasmin inhibitors.

**Fig. 9:**
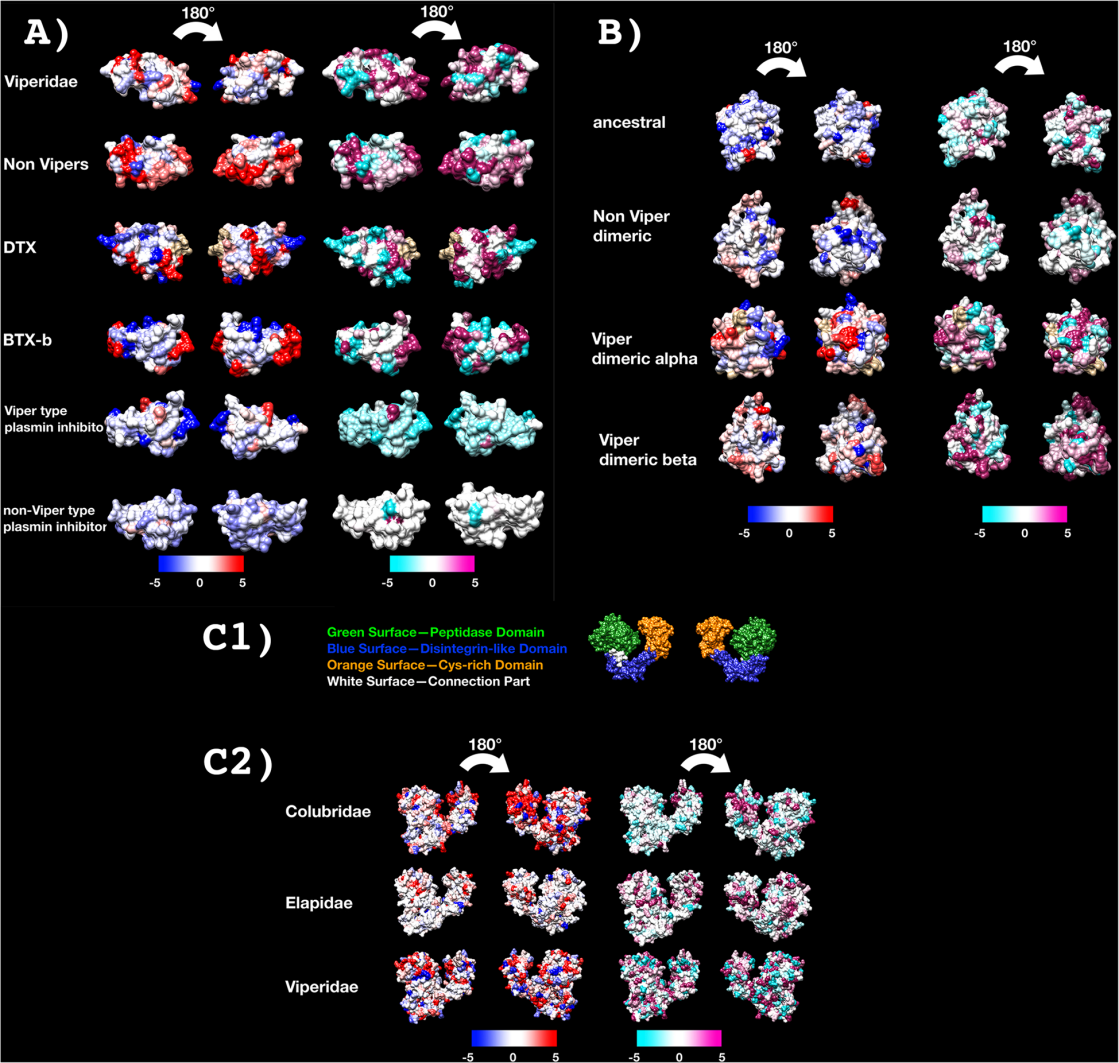
Molecular modelling of showing sites under selection by FUBAR (left) and MEME (right) colour coded to show sites that are negatively (blue and turquoise, resp), neutrally (white), or positively (red and purple resp.) selected. Protein models show front and back views coloured according to FUBAR’s estimated strength of selection (*β*-*α*, left) and MEME’s significance levels (right). See Tables 1 and 2 for values. See Additional file 4 for modelling templates. **A** Kunitz, **B** lectin, **C** snake venom metalloproteases (SVMP) coloured to show domain locations (**C1**) and sites under selection (**C2**)

Selection analyses revealed very different rates of molecular evolution for the kunitz toxin type within the snake families (Fig. 7 and Table 1), strongly suggesting that there is a multiplicity of undocumented novel activities yet to be discovered across the full range of this toxin class. This is consistent of the documentation of three evolutions of neurotoxin function within just the elapid snakes. The differential rates between the monomeric dendrotoxins (*ω* = 2.10) and the disulphide-linked β-bungarotoxin subunits (*ω* = 1.23) is consistent with the structural constraints imposed upon the β-bungarotoxin subunits by being not only part of a multi-subunit complex, but a disulphide-linked one at that. However, despite these structural constraints, the β-bungarotoxin subunits display evidence of individual sites under positive selection. In contrast to the neurotoxins, but consistent with the high structural conservation of the enzymatic pathophysiological target, both independent lineages of plasmin inhibitors are under negative purifying selection pressures (Fig. 9A and Table 1).

### Lectins

**Fig. 10:**
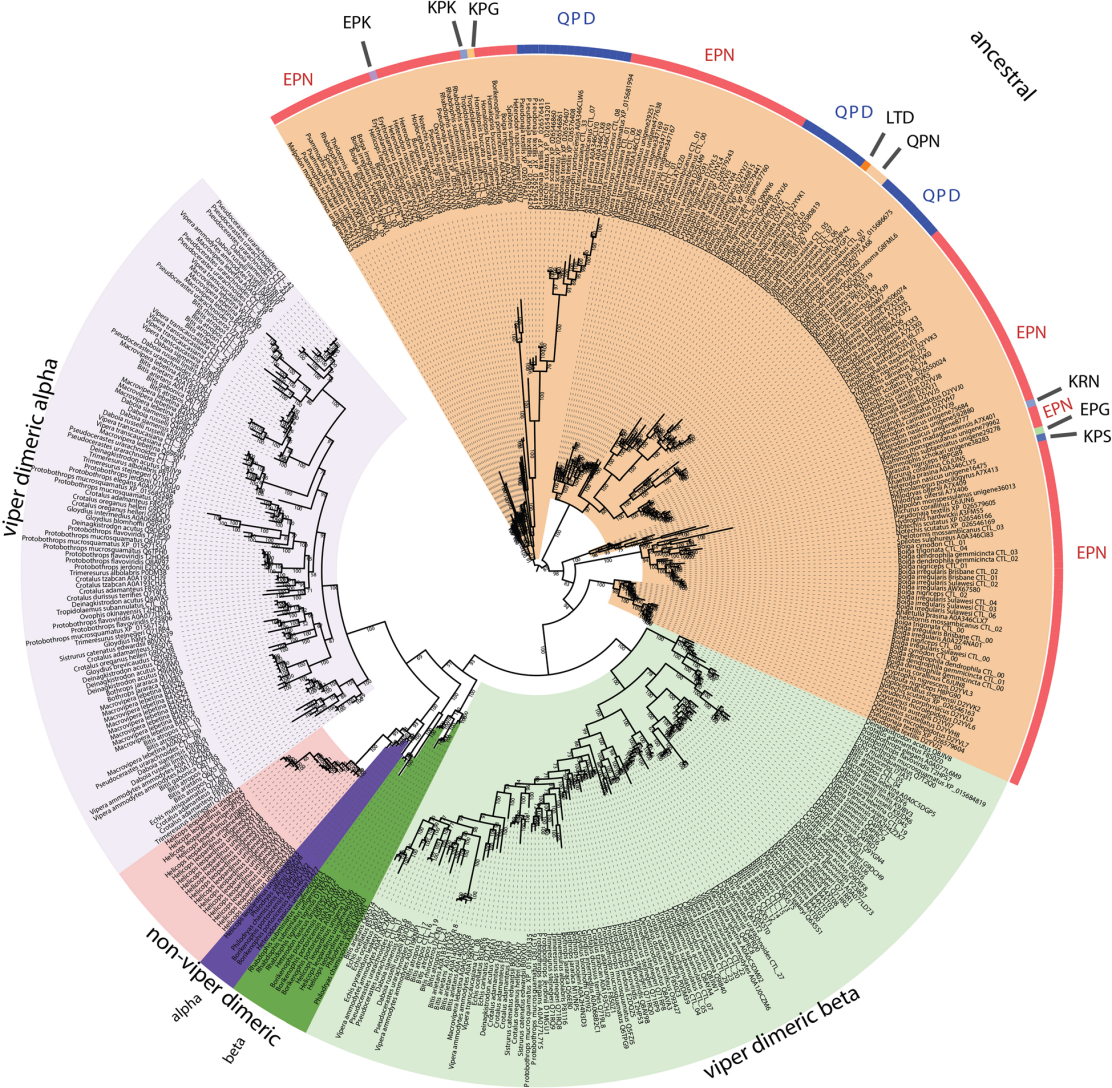
Molecular phylogenetic reconstruction of lectin toxins. The ancestral monomeric form is shaded annotated with variations in the key functional amino acid triad. Additional files contain sequence alignment used for constructing phylogenetic tree (Additional file 17), tree output file (Additional file 18), and zoomable high-resolution tree image (Additional file 19)

**Fig. 11:**
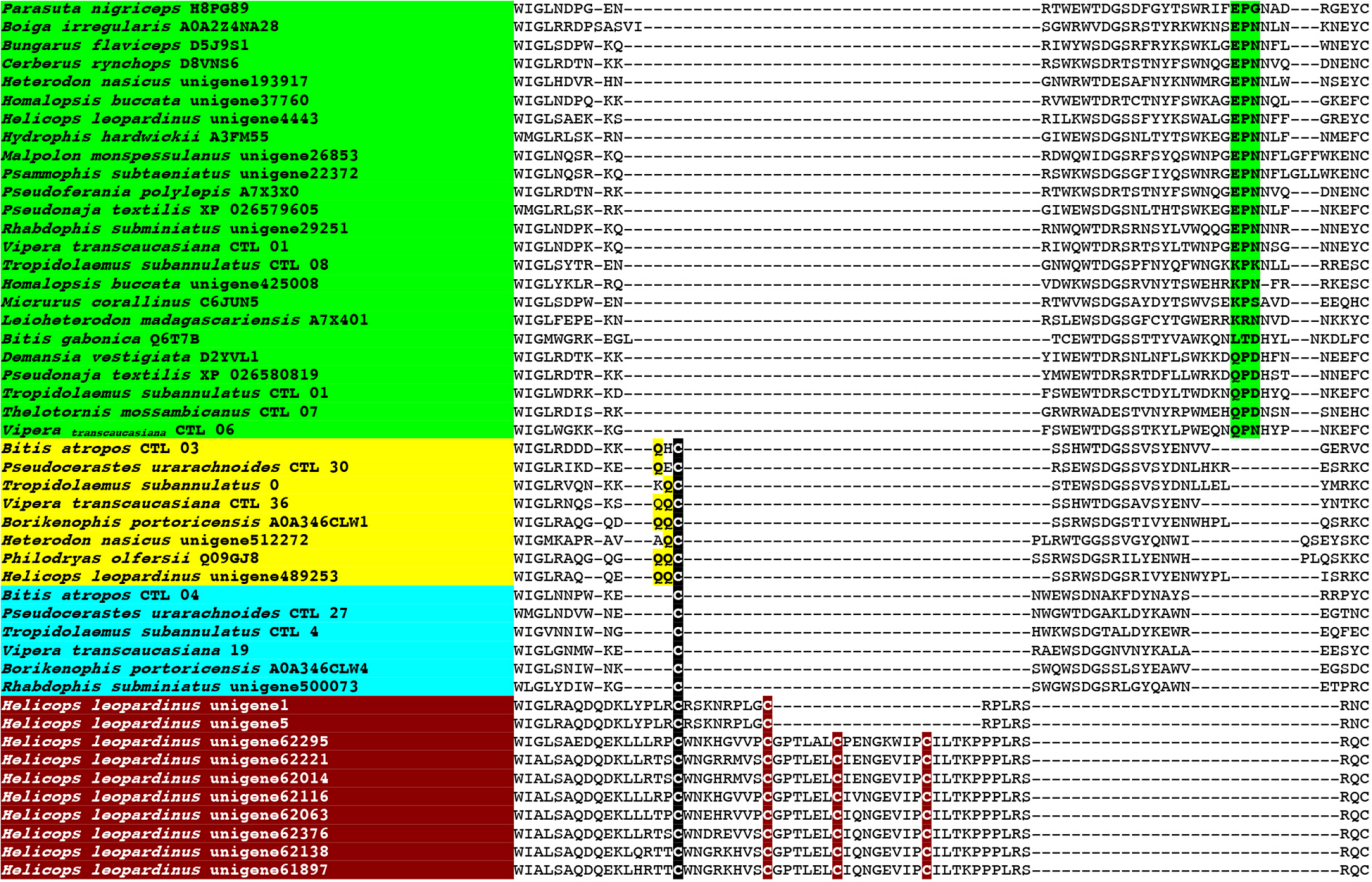
Partial sequence alignment of representative lectin toxins in the region that contains the key functional amino acid triad (shaded in green). The alpha chain of the derived cysteine-linked heterodimeric form is shaded in yellow, including the glutamine motif that diagnoses the alpha chain, while the beta chain is highlighted in blue. The newly evolved dimer-forming cysteine is shaded in black. The sequence alignment also shows the insertion characteristic of an extremely derived form known currently only from *Helicops leopardinus*, which contains unique cysteines shaded in burgundy

The basal form of lectin toxins in reptile venoms is a single-chain form which may form non-covalently linked complexes and contains diagnostic tripeptide functional motif (Figs. 10 and 11) [98, 99]. Consistent with previous analyses [2], our phylogenetic results suggest that the basal functional motif is the amino acids EPN (glutamic acid + proline + asparagine). Our results also indicate that the QPD (glutamine + proline + aspartic acid) motif has arisen on two convergent occasions, once in the last common ancestor of the advanced snakes, and again in the last common ancestor of the Australian radiation of elapids (Figs. 10 and 11). In addition, other mutations in the functional motif were documented across a myriad of lineages: EPG (glutamic acid + proline + glycine) in *Parasuta nigriceps* within the Elapidae; EPK (glutamic acid + proline + lysine) in *Heterodon nasicus* within the Colubridae; KPK (lysine + proline + lysine) in *Tropidolaemus subannulatus* within the Viperidae; KPN (lysine + proline + asparagine) in *Homalopsis buccata* within the Homalopsidae; KPS (lysine + proline + serine) in *Micrurus corallinus* within the Elapidae; KRN (lysine + arginine + asparagine) in *Leioheterodon madagascarensis* within the Lamprophiidae; LTD (leucine + threonine + aspartic acid) in *Bitis gabonica* within the Viperidae; and QPN (glutamine + proline + asparagine) in *Vipera transcaucasiana* within the Viperidae (Figs. 10 and 11). To date, only viperid venom variants of the QPD form have had their bioactivity tested and were shown agglutinate erythrocytes and promote edema by increasing vascular permeability [100–102]. The impacts of the extreme diversifications of the key functional motif shown in this study upon the functions of the toxins are entirely unknown and require further research. The overall *ω* value for these toxins was only 0.72, but there were 14 sites identified as positively selected by FUBAR and MEME which is an indication that the variation which occurs in these toxins is tightly constrained and only occurs at a relatively small subset of positions (Table 1).

In addition to the ancestral single-chain form, a disulphide-linked dimer composed of two different lectins has long been known from viperid venoms (sometimes referred to as snaclecs, or C-type lectins), with variants producing a wide diversity of coagulotoxic effects including inhibition of the clotting factor vWF and clotting enzymes such as Factors IXa and XIa [99]. In addition to the diagnostic newly evolved cysteines that facilitate the inter-chain disulphide bond leading to the dimeric tertiary structure, this type is also molecularly distinct because they have lost the functional motif (Fig. 11). Instead they exert their neofunctionalised toxicities through novel binding sites. The *α* and *β* chains have a newly evolved inter-chain cysteine in the same position which suggests that either these toxins evolved from a single gene that produced a homomeric ancestral toxin and subsequently underwent duplication and subfunctionalization or that structural constraints led to the novel cysteine mutation occurring at the same location in two different genes. The *α* chain is readily distinguished from the *β* chain by a characteristic glutamine motif present immediately before this cysteine that diagnostic cysteine (Fig. 11). In this study, not only did we recover multiple variants from non-viperid snakes that possessed the diagnostic cysteine of the dimeric lectin form, but forms with (alpha chain) and without (beta chain) the glutamine motif form were present (Fig. 11). Thus the evolution of the dimeric lectin toxins preceded the divergence of Viperidae from other advanced snakes. However, the rates of evolution are reflective of the explosive diversification of this toxin type within the viperids (Fig. 9B and Table 1). Consistent with a previous report of differential rates of evolution between the *α*-subunit and *β*-subunits looking at the venom of just one species (*Crotalus helleri*) [103], we found that the *α*-subunit and *β*-subunit were subject to different selection pressures when analysed across all viperid species. The *α*-subunit had an overall *ω* value of 1.40 with 49 sites shown to be positively selected by FUBAR and MEME, while the *β*-subunit had an overall *ω* value of 1.27 with 38 sites shown to be positively selected by FUBAR and MEME (Table 1). In contrast, the non-viperid dimeric forms (non-viperid alpha and. beta sequences combined into a single set due to low numbers of overall sequences but excluding the unique diversification within *Helicops*) had an overall neutral *ω* of 0.97 but with 10 sites shown as positively selected by FUBAR and MEME (Table 1). The comparative analysis of 3D modelling (Fig. 9B) on different clades showed that those residues under positive selection are located on different position of the surface for the viperid *α*-subunit versus *β*-subunits, and the other forms as well, indicative of different selective forces and the potential for the discovery of novel activities within the non-viperid dimeric and monomeric forms. Structure-function studies on these toxins may therefore be particularly interesting. The unique form present in the venom of *Helicops leopardinus*, which has novel insertions in the key functional region, including the evolution of novel cysteines may also be of particular interest for these future research efforts (Fig. 11).

### Snake venom metalloproteinases (SVMPs)

**Fig. 12:**
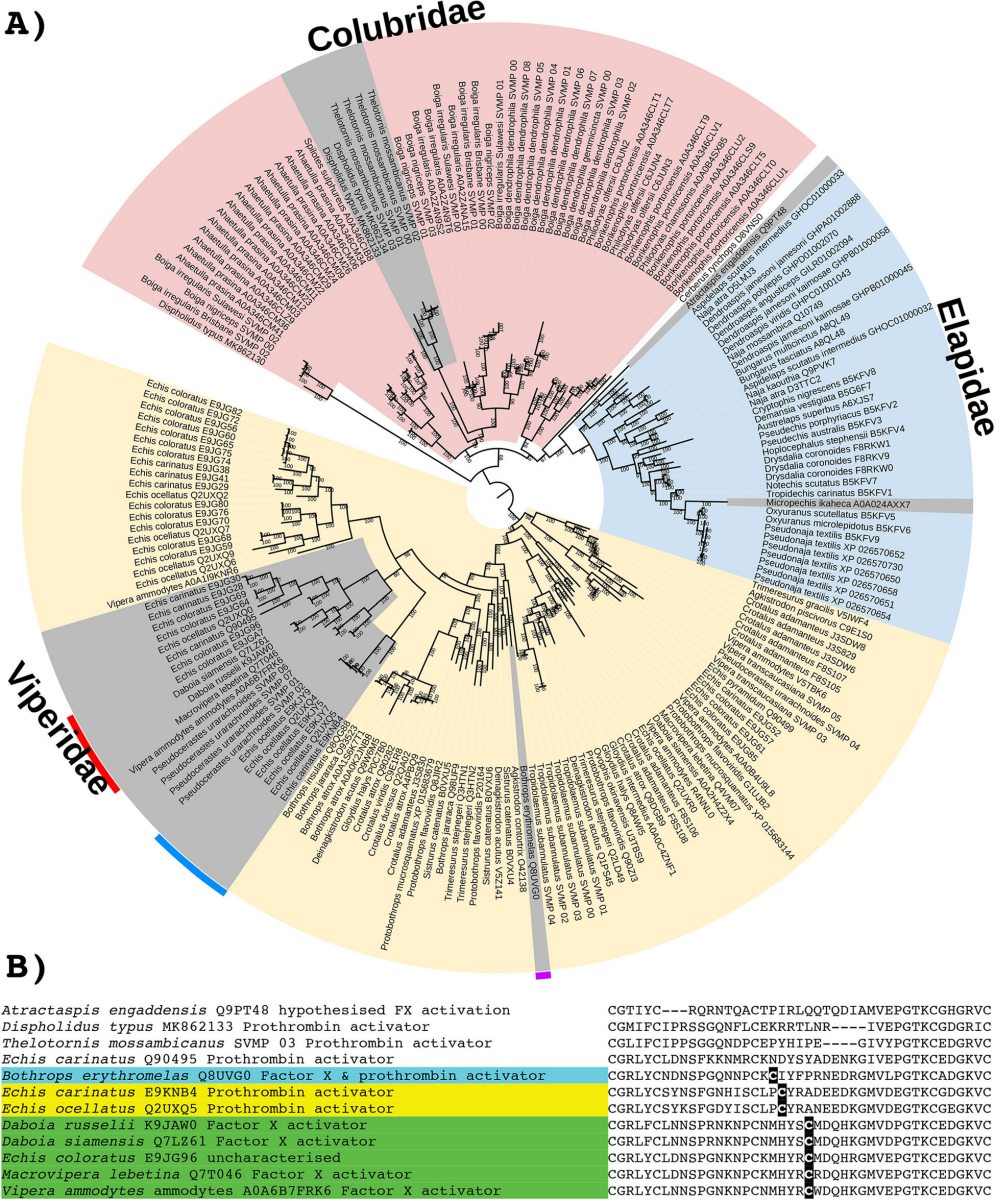
**A** Molecular phylogenetic reconstruction of SVMP toxins with the lineage-specific amplification of particular forms shaded in pink (Colubridae), blue (Elapidae), or yellow (Viperidae). Grey shading shows the convergent evolutions of procoagulant functionally derived forms. Colours on the outside of the ring designate the convergent evolutions of the P-IIId structurally derived forms. Additional Files contain sequence alignment used for constructing phylogenetic tree (Additional file 20), tree output file (Additional file 21), and zoomable high-resolution tree image (Additional file 22). **B** Partial sequence alignment of representative SVMP with coloured shading of species names indicating the three convergent evolutions of inter-chain cysteines diagnostic of the P-IIId structurally derived forms. Other procoagulant functionally derived forms (species names not shaded) are included for comparison

While SVMPs have long been known as one of the dominant venom types in viperid venoms, increasing evidence is emerging of their importance in the venoms of other families [2, 73, 104–108]. The basal SVMP structural form (P-III) is a final processed protein consisting of three domains: protease + disintegrin + cysteine-rich. Domain deletion forms are largely known only from viperid venoms which include the P-II (protease + disintegrin, with the cysteine-rich domain deleted) and P-I (protease only, with both the disintegrin and cysteine-rich domains deleted) [106]. Intriguingly the P-I derived condition appears to have evolved convergently in the dipsadinae lineage within the Colubridae rear-fanged snake family [109]. The P-III form, however, remains a major constituent of viperid venoms, and other than the select dipsadinae lineages referred to in the above sentence, it is the only form present in non-viperid snakes. Consistent with the structural and functional diversification within the viperids, phylogenetic analysis in this study revealed evidence of extensive gene duplication in the last common ancestor of the viperid snakes (Fig. 12). In contrast, for the colubrid and elapid snakes, the sequences broadly follow organismal relationships, with diversification events largely confined within a particular lineage.

Within the P-III SVMP enzymatic toxin class, there have been convergent structural derivations characterised by the evolution of a new cysteine that allows these toxins to form covalently linked multimers with lectin dimers. SVMP with this novel cysteine are termed P-IIId. Phylogenetic analysis suggests that the P-IIId type have evolved on at least three occasions: *Bothrops*; the last common ancestor of *Daboia/Macrovipera/Montivipera/Vipera*; and again in *Echis* (Fig. 12). Our phylogenetic analyses (Fig. 12) demonstrates that *Echis* venoms contain two distinct forms of P-IIId (one unique to the genus and one shared with *Daboia/Macrovipera/Montivipera/Vipera*), which confirms previous hypotheses that were based on sequence similarity calculations but did not undertakephylogenetic analyses [110]. Sequence analysis in this study shows that while the cysteines have evolved in homologous regions of the SVMP scaffold, suggesting structural constraints in the formation of a multimeric complex, they differ slightly in position and, consistent with independent evolutions, differ in flanking residues (Fig. 12). The *Daboia/Macrovipera/Vipera* P-IIId are selective for the activation of factor X, and these venoms do not additionally activate prothrombin [111–113]. In contrast, while both *Bothrops* and *Echis* crude venoms activate factor X in addition to activating prothrombin [114, 115], the relative roles of P-IIIa versus P-IIId individual toxins in activating the two clotting factors remains to be elucidated.

In addition to structural diversifications, SVMPs have acquired a number of novel functions, the most common of which is procoagulant activity [106]. Identifying sequences in our phylogeny that have demonstrated procoagulant effects suggests that within the viperids the procoagulant trait has evolved independently within the viperine subfamily (such as *Echis*, *Daboia*, *Macrovipera*, *Pseudocerastes*, and *Vipera*) and again in the crotaline subfamily (*Bothrops *genus) (Fig. 12). Clotting factor activation has also been documented in the additional crotaline genera *Calloselasma* (Factor X) and *Crotalus* (Factor X by neonate *Crotalus* *culminatus*) [116, 117], but the toxins responsible have not been sequenced. Consequently, their phylogenetic affinity to the *Bothrops*-type procoagulant P-III SVMP is unknown, and it cannot be determined whether procoagulant SVMPs have evolved once or several times in the pit vipers. Once the sequences become available, this will be resolved by whether the toxins form a monophyletic group with the *Bothrops* toxins (thus suggesting an early evolution with this trait amplified on multiple convergent occations) or if they form distinct clades (thus suggesting convergent neofunctionalisation). In addition to evolving at least twice within the viperids, the procoagulant SVMP trait evolved independently again in the last common ancestor of the colubrid genera *Dispholidus* and *Thelatornis* [118] and also in the elapid genus *Micropechis* [119], with the toxins being phylogenetically distinct from viperid forms (Fig. 12). If P-III SVMP are responsible for the procoagulant activity shown for *Atractaspis* venoms [120], then this would represent another convergent evolution of this trait as the *Atractaspis* P-III SVMP are also phylogenetically distinct from viper procoagulant forms (Fig. 12). Similarly, the toxins responsible for the procoagulant toxicity of the *Rhabdophis* genus have not been identified [121, 122], but if the *Rhabdophis* procoagulant effect is due to a SVMP, this would almost certainly represent another instance of functional convergence considering the tens of millions of years of separation between this genus and the other procoagulant lineages.

The overall *ω* value for all lineages was consistently higher for the cysteine-rich domains than in the disintegrin or protease domain. This suggests that the cysteine-rich domain is crucial for target binding prior to the interaction of the catalytic site located on the protease domain, and therefore, this is a critical domain for the evolution of neofunctionalisation. Analysis of selection (Table 2) and 3D modelling (Fig. 9C) showed that more than half of the positively selected sites detected were confined to the protease domain and of the remaining variations more were found in the cysteine-rich domains than the disintegrin-like domains. Again, this pattern suggests a bias in positive selection toward the protease domain, consistent with this being the subunit responsible for the enzymatic activity.

**Table 2:** Molecular evolutionary rates of SVMP (See Fig. 9C for modelling)

Clade	Domains	***ω***	FUBAR (−)^**a**^	FUBAR (+)^**b**^	MEME^**c**^	FUBAR and MEME^**d**^
**Colubridae**	**Full-length secreted form**	1.19	74	103	46	33
	**Peptidase domain**	1.54	19	53	50	39
	**Disintegrin domain**	0.72	s	12	12	10
	**Cys-rich domain**	1.64	18	30	36	24
**Elapidae**	**Full-length secreted form**	1.23	36	75	86	56
	**Peptidase domain**	1.47	13	25	35	21
	**Disintegrin domain**	0.86	5	8	9	7
	**Cys-rich domain**	1.76	7	29	36	21
**Viperidae**	**Full-length secreted form**	1.37	79	132	159	124
	**Peptidase domain**	1.44	22	57	78	55
	**Disintegrin domain**	0.80	23	21	21	18
	**Cys-rich domain**	1.63	27	41	50	38

^a^Number of codons under negative selection according to FUBAR

^b^Number of codons under positive selection according to FUBAR

^c^Number of codons under episodic diversifying selection according to MEME

^d^Number of codons that fit criteria ^b^ and ^c^

### SVMP propeptide domain novel toxins

**Fig. 13:**
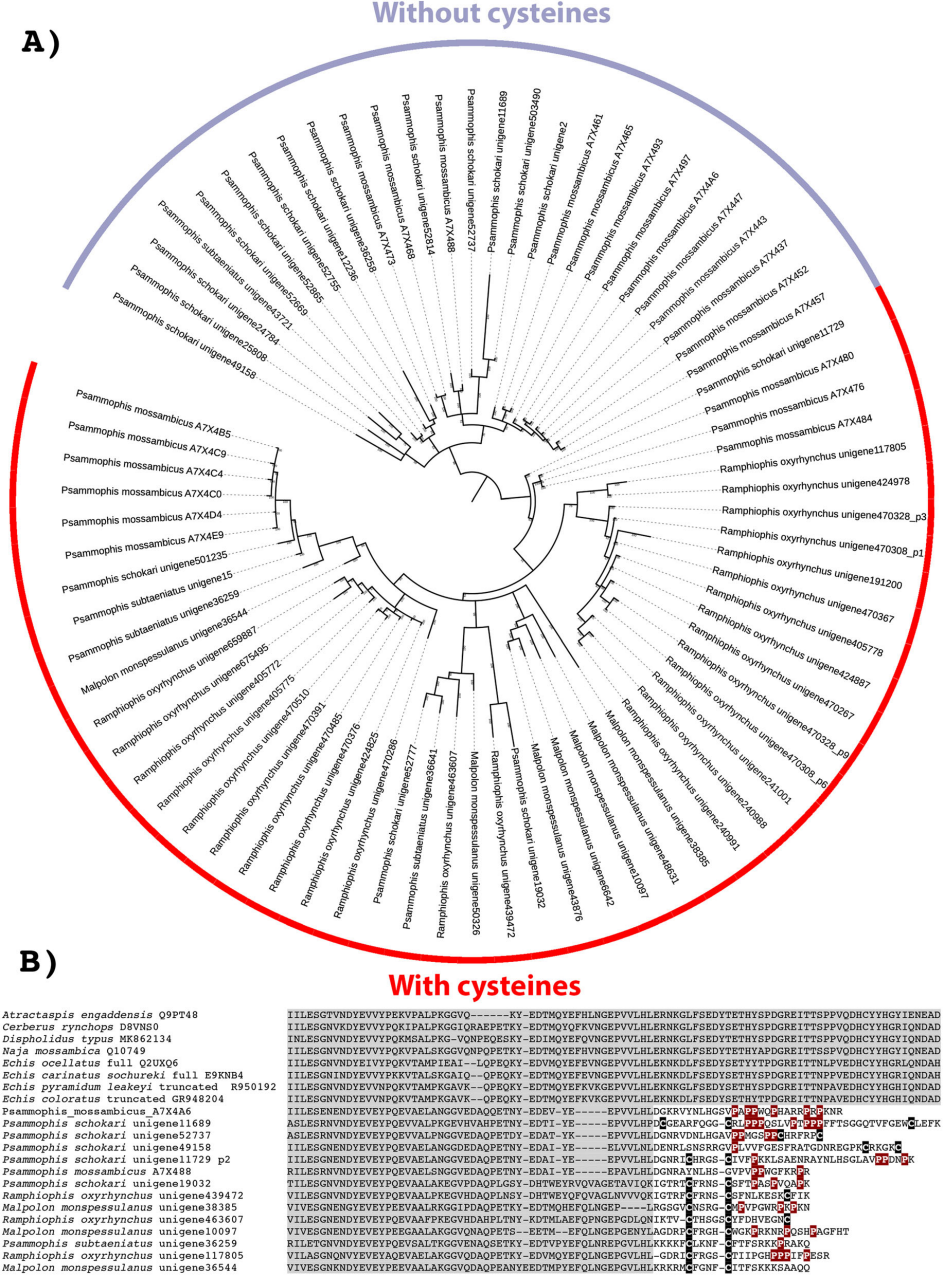
**A** Molecular phylogenetic reconstruction of the psammophiine lineage-specific derivation of the SVMP propeptide domain into a novel toxin family, with the subsequent explosive diversification of the cysteine-linked forms. Additional Files contain sequence alignment used for constructing phylogenetic tree (Additional file 23), tree output file (Additional file 24), and zoomable high-resolution tree image (Additional file 25). **B** Alignment of representative SVMP proproptide domains. Region shaded in grey is that which is homologous across all representatives, with the unshaded region indicating the location of the putative frame-shift mutation that led to the evolution of the new toxin family in psammophiine snakes. Cysteines are highlighted in black, secondary structure imparting prolines highlighted in red

In addition to the structural variations noted in the above section, on at least two independent occasions the propeptide domain of SVMP genes have been recruited as toxins in their own right, without accompanying expression of any of the three domains making up the P-III enzyme. This was first noted in *Echis* venoms, where the truncation is formed by stop codons terminating otherwise unremarkable sequences [123]. More intriguing toxins are found in the venoms of psammophiine snakes which were first noted in the species *Psammophis mossambica* [2], where the propeptide domain was selectively expressed. Unlike the *Echis* forms, there was explosive diversification of these novel toxins: 26 variants were discovered in this species alone, including forms with novel cysteines which could potentially form disulphide bonds. Subsequent testing of two of these toxins revealed them to be novel neurotoxins [65]. The activity of the other variants is unknown. In this study, this novel toxin class was shown to be present with staggering sequence diversity across the psammophiine snakes, including not only the additional *Psammophis* species we sequenced but also the *Malpolon* and *Rhamphiophis* species (Fig. 13). Sequence analysis revealed that the first half of the toxins are homologous to the propeptide region of typical P-III genes (Fig. 13). However, there is then an abrupt shift in sequence patterns, which is consistent with a frame-shift mutation providing the starting substrate for the evolution of this novel toxin class. The subsequent evolution resulted in such sequence diversity that calculating rates of evolution was impossible due to the unalignable diversity in the second half of the peptides. Such incredible diversity suggests there may be extensive neofunctionalisation beyond the previously characterised neurotoxicity. Therefore, this toxin class represents a particularly rich area for future research, especially as most of these toxins are either short linear or with a single disulphide bond, which would allow for efficient synthesis.

## Discussion

The findings of this study represent a significant increase in our knowledge of the broad-scale molecular evolution of these snake toxin families. We have revealed novel patterns of expression of basal toxin types, including previously unrecognised instances of molecular and structural convergence. The new toxin encoding sequences that we have included in our analyses proved particularly valuable for demonstrating the distribution of novel toxin classes derived from the propeptide domains of pre-existing toxin genes. These results provide a framework to help guide future bioactivity testing work and further evolutionary studies. Research into the evolutionary and selective forces that result in the instances of explosive diversification or molecular convergence will provide crucial insights into how venom evolves.

One interesting question raised by these data is why, despite all these toxin families being present in the last common ancestor of the advanced snakes, particular descendant lineages have specialised into the production and refinement of certain toxin families and isoforms. For example, kallikrein enzymes with the ability to destructively cleave of fibrinogen seem to have already evolved and been present in the last common Toxicofera reptile ancestor [1, 50, 70, 84, 87, 88, 124, 125]. However, despite being such a basal trait, these toxins have only been amplified to form a major part of the venom on two convergent occasions: in the Anguimorpha lizards and the viperid snakes (Fig. 8). Conversely, 3FTx have undergone relatively little molecular evolution within viperids, while the basal form has been extensively structurally modified in rear-fanged lineages (including the formation of dimeric forms in colubrids) and are the dominant toxin family in some lineages. Meanwhile, the elapids have predominantly exploited the derived forms which lack the 2nd and 3rd ancestral cysteines with much less gene duplication or expression of the basal 3FTx. In contrast,kunitz peptides are fairly common in viperid venoms and have diversified to an extreme degree in the elapid family, but are largely absent in other lineages. It is unclear whether these lineage-specific differences are the product of chance or if they were constrained by the ecological contexts in which the progenitors of these snake families employed their venoms.

Other toxin types—such as the SVMPs—show broadly more similar levels of duplication across multiple snake families. This mirrors the pattern of neofunctionalization, such as P-III SVMPs mutating into potent procoagulant factor activating toxins on multiple convergent occasions. However, only in the viperids has the P-IIId multimeric form evolved, where a SVMP is covalently linked to a lectin dimer (which already possesses another inter-chain disulphide bond). The viperids are by far the lineage with the greatest diversity of lectin dimers which may have provided a greater range of molecular opportunities for the P-IIId trait to evolve. As with the various subtypes of 3FTx, the success of dimeric lectins in viperids is associated with a relative paucity of sequence diversity and expression for the basal monomeric isoforms which have, in turn, undergone much more duplication in other snake lineages, showing extensive variations in the key functional motif. The CRiSP toxins represent yet another evolutionary pattern, where they are fairly diverse across the lineages, but with different isoforms forming major clades in each.

From a broad perspective, almost all toxin types we examined demonstrate a phylogenetic pattern of large clades belonging to snakes of the same family (Figs. 1, 3, 5, 6, 7, and 12). This suggests that these toxins had not yet extensively diversified in the common ancestor of Colubroidea. The only exception was the lectins (Fig. 10), which suggests that this common ancestor likely possessed multiple copies of this toxin already including the derived dimeric forms. Duplication of toxin genes in snakes is often associated with higher abundance of that toxin family in the final venom composition [126, 127], and with duplication being evident by sequence variation due to errors introduced during gene copying events, so this may constitute preliminary evidence that the common ancestor of Colubroidea would have possessed a venom with high lectin composition paralleling the venom system innovations such as partitioned oral glands and perhaps modified dentition. The pattern we see in the other families indicates that the vast majority of the variation in terms of composition, unique structures, and novel functions that we observe in extant snake venoms arose after the divergence between the families and during the diversification and specialization of those lineages.

While we have discussed many toxins which have rapidly diversified, this phenomenon is at its most extreme in the propeptide regions of the natriuretic and SVMP genes. Typically, the propeptide regions of these toxins types are post-translationally cleaved but not secreted and consequently do not play roles in envenomation. However, in both these families, new mutations have caused part of the propeptide region to be translated into post-translationally liberated and secreted peptides to form entirely new toxin classes with novel functions. For the natriuretic peptides, the newly evolved toxins include repeating series of bradykinin-potentiating peptides which increase the hypotensive effect of the venoms [48]. The viperid genera *Azemiops* and *Tropidolaemus* have separately evolved novel neurotoxins derived from within the natriuretic propeptide domain. In both cases, despite their independent origins they share the unusual feature of creating multiple peptides that are translated from a single transcript and then post-translationally liberated. Thus the natriuretic gene encodes for multiple discrete products in the form of the natriuretic peptide and the various *de novo* evolved peptides from within the propeptide region. Another peptide type was first documented in *Dendroaspis* venom and the molecular evolutionary history remained enigmatic, but our analyses indicate these are members of yet another novel toxin class arising from the natriuretic gene propeptide region. The most explosive diversification of all toxin classes was that of the newly evolved toxin family that evolved in the SVMP propeptide domain within psammophiine snakes. The staggering sequence and structural diversity of these toxins makes it likely that other toxic activities in addition to the already documented novel neurotoxic forms [65] will be documented as more bioactivity testing is undertaken.

Our analyses show that these shared colubroid toxin families exhibit remarkable instances of convergent evolution in terms of pathophysiological neofunctionalisation and derived protein structures. For example, within two potently procoagulant lineages (the *Daboia* genus within the viperid snakes and the *Oxyuranus/Pseudonaja* clade within the elapid snakes), plasmin-inhibiting kunitz peptides have evolved which would potentiate the procoagulant effects by increasing the half-life of the blood clots formed due to the inhibition of the blood clot destroying enzyme plasmin. Other taxa possess the arginine residue that is crucial for these plasmin inhibitors and may represent further instances of convergence if functional research confirms this hypothesised activity. Similarly, within the SVMP neofunctionalised procoagulant variants which activate Factor X or prothrombin have arisen on multiple independent occasions. The lectins may potentially contain further examples of functional convergence within a toxin type given the multiple origins of the QPD motif at a key functional location, but these have not been bioactivity tested.

One of the most striking cases of structural convergence is the previously mentioned P-IIId derived form of the SVMP which form a covalent linkage to a lectin dimer. The novel cysteine crucial for the formation of these toxin complexes was shown to have evolved on at least three separate occasions within the viperids as structural modifications of forms that were themselves functionally derived (procoagulant). The selection pressures leading to this convergence have not been explored and the functional impacts (Factor X versus prothrombin activation) are similarly uncharacterised. This is similar to the evolution of covalently linked dimers of basal-type 3FTx. Within the Colubridae toxins with the derived elongation of N-terminus and propeptide domain, *Boiga* and *Spilotes* evolved novel cysteines in two different 3FTx each which allowed for the formation of heterodimers; most remarkably, these cysteines appear to have independently evolved at the same position in the two genera. The dimeric lectins may have had a similar coincidence in their evolution: both the *α-* and *β*-subunits possess novel cysteines at the same residue, but it is unclear if these cysteines are truly convergent or if the two subunits are the result of a duplication of an ancestral lectin which already possessed a cysteine at that site. The natriuretic peptides also demonstrate convergent structural evolution in the repeated origins of variants with C-terminal tails as well as the previously discussed independent neurotoxins derived from the propeptide region.

The kunitz peptides have been the substrate for both levels of convergence (structural and functional). Structurally three out of the four neurotoxin types (MitTx, taicatoxin, and bungarotoxins) converge in their formation of heteromers with PLA_2_ subunits, but diverge structurally in this regard by being non-covalently linked (MitTx and taicatoxin) or covalently linked (β-bungarotoxin), and also in the number of PLA_2_ subunits associated with (MitTx = 2, taicatoxin = 1, β-bungarotoxin = 1). All four of the neurotoxic kunitz peptides converge in being ion channel toxins, with dendrotoxins and β-bungarotoxins further converging on the same target (*K*_V_ channels). While taicatoxins affect a different ion channel type (L-type calcium channels) than dendrotoxins and β-bungarotoxins, they converge with bungarotoxin in the PLA_2_ toxin facilitating a secondary action that results in the net functional outcome in blocking the release acetylcholine, leading to flaccid paralysis. In contrast, dendrotoxins act upon the voltage-gated potassium channels to facilitate acetylcholine release, leading to spastic paralysis. β-bungarotoxin demonstrates another instance of convergent molecular evolution in the novel cysteines which allow for the formation of these complexes have evolved twice in the exact same location within this genus. The convergent evolution of novel cysteines at the same residue on two occasions within both β-bungarotoxin and the 3FTx dimers is strongly indicative of structural constraints in dimer formation.

## Conclusion

This study gives a broad overview of the structural and functional diversity in the toxin families which are homologous in colubroid snake venoms. It is this diversity that produces the wide range of clinical effects and variable responses to antivenom that contribute to the global problem of snakebite. However, such molecular diversity also provides fertile ground in the search for novel molecules as lead compounds for the discovery of new tools and medications. This diversity has also allowed these toxins to converge repeatedly on similar sequences, structures, and functions. This widespread convergence suggests that certain pathophysiological activities and certain configurations of proteins may be evolutionary ‘good tricks’ [128] that are similarly effective across multiple taxa and may solve evolutionary problems that venoms encounter such as prey resistance.

## Methods

### Species studied

Transcriptomes were constructed for the following families and species: Colubridae - *Helicops leopardinus*, *Heterodon nasicus*, *Rhabdophis subminiatus*; Homalopsidae – *Homalopsis buccata*; Lamprophiidae - *Malpolon monspessulanus*, *Psammophis schokari*, *Psammophis subtaeniatus*, *Rhamphiophis oxyrhynchus*; and Viperidae – *Bitis atropos*, *Pseudocerastes urarachnoides*, *Tropidolaeumus subannulatus*, *Vipera transcaucasiana.* Glands from euthanised captive specimens (one per species) were obtained under University of Melbourne Animal Ethics Approval UM0706247-2005 and University of Queensland Animal Ethics Approval 2021/AE000075. A *Pseudocerastes urarachnoides* tissue sample was provided by PG and BF under Iranian approval NIMAD # 942485.

### Transcriptome sequencing and assembly

Total RNA was extracted with Trizol reagent (Invitrogen, Carlsbad, CA, USA) and purified using RNeasy Animal Mini Kit (Qiagen, Valencia, CA, USA). The construction of cDNA libraries and RNA-seq were performed as previously reported [129]. In brief, the poly-A containing mRNA molecules were purified using poly-T oligo-attached magnetic beads, then separated from the total RNA by Oligo (dT) and fragmented into small pieces randomly using divalent cations under elevated temperature. The cleaved RNA fragments were copied to synthesise the first-strand cDNA using reverse transcriptase and random hexamer primers, then the second-strand cDNAs were synthesised with the buffer, dNTPs, DNA polymerase I, and RNase H (Takara Biotechnology, Beijing, China). After synthesis, these cDNA fragments were ligated with the adapters, then purified, and the final cDNA libraries were constructed by PCR enrichment. After the construction of cDNA libraries, Qubit 2.0 and Agilent 2100 were used for preliminary quantification and detecting the insert size of the libraries respectively. After passing the screening, the qualified cDNA libraries were sequenced through Illumina Hiseq X ten and BGISEQ-500 at BGI (Shenzhen, China) with 150 bp paired-end reads. The Illumina HiSeq X Ten sequencing platform generated 56.34~73.44 M raw reads from nine samples and the BGISEQ-500 sequencing platform generated 177.32 M raw reads from *R. oxyrhynchus*. Raw sequencing data were deposited in the Short Read Archive (SRA) of the NCBI with accession numbers of SRR12802473~SRR12802481 and SRR13234020 (Additional fle 26). After trimming out the low-quality reads with Fastp [130], 55.17~71.18 M clean reads were generated from 9 samples respectively and 174.16 M clean reads from *R. oxyrhynchus*. Of these clean reads, the Q30 percentage in each library was approximately 90%, which indicated good quality of sequencing (Additional file 4). Transcriptome sequencing of *B. atropos* and *T. subannulatus* were from Sanger sequencing as previously described [107].

Decontamination was undertaken by identifying *k*-mers (length set by *-k*, recommended value 57) in our focal read set that were present in another read set from the same lane at a higher level (x-fold shift set by *-d*, recommended value 1000). Reads with a certain percentage (set by *-p*, recommended value 0.25) of their sequence represented by such *k*-mers were filtered from the data set. Raw reads were checked for potential sample cross-leakage through index mis-assignment within the same sequencing lane. Counts of all 57-mers in the raw reads for each sample in each lane were generated with Jellyfish v. 2.2.6 [131], and 57-mers that showed > 1000× count differentials between each pair of samples in a lane were identified. Reads with 25% or more of their lengths comprised of 57-mers in this set were removed from the sample with lower counts.

We used Fastp v. 0.20.0 [130] for adapter and quality trimming. Paired forward and reverse reads were overlapped into longer single-end reads with PEAR v. 0.9.11 [132] as input for assembler Extender. Different assemblers have different strengths and weaknesses. Our strategy is to make several different assemblies and resolve the data later with our quality control methods. To compare assembly methods, we chose several assemblers which have been widely used for *de novo* transcriptome assembling, and assembled the same short-read RNA-seq data with each assembler.

Two of these assemblers used variations of the *de Bruijn* graph approach to contig construction: SOAPdenovo-Trans [133] and Trinity [134]. We also employed BinPacker [135], an assembler that incorporates coverage information to construct its splicing graphs and is reported to perform well with multi-isoform data. Finally, we used an in-house assembler, Extender [136], which randomly selects seed reads and extends these outward based on matching overlap with other reads to form contigs. The VTBuilder [137] assembler implements a similar seed-and-extension algorithm in its approach to multi-isoform transcript assembly. However, its current limit of five million input reads renders it unsuitable for the current scale of RNA-seq datasets (59~73 million reads per sample for our data), and we therefore did not evaluate it. Assembly methods differed in the format of input reads and therefore in overall read counts used for each assembly. In this study, BinPacker, SOAPdenovo-trans, and Trinity processed with paired pairs, whereas Extender processed with the merged single pairs.

SOAPdenovo-trans differs in that it requires a config file for settings. It is also unique in that the results vary enough based on *k*-mer size so we run it with a range of different *k*-mer sizes. We ran SOAPdenovo-trans v. 1.03 at five different *k*-mer sizes: *k* = 25, *k* = 31, *k* = 75, *k* = 95, and *k* = 127 with each run saved as an independent assembly. Maximum and minimum read lengths were set at 500 and 200 bp, respectively, and average insert size was set at 250 bp. For Trinity assembly, we ran Trinity v. 2.5.1 with a minimum contig length of 150 bp and a *k*-mer of *k* = 25. BinPacker v. 1.0 was similarly run with a *k*-mer size of *k* = 25. Finally, we ran our only seed and assemble approach—Extender [136], with 2000 randomly selected seeds with a minimum quality of 30 at all base positions. Seeds were prohibited from sharing any *k*-mers as long as the extension-overlap length (100 bp). We required at least two extensions for each direction to retain the results of the seed. We set a 100-bp minimum overlap for extension and a minimum quality score of at least 20 at all base positions for a read to be considered for extension. We allowed 20 replicates per seed per direction and required that 20% of replicates per seed be extended in order retain a seed. In order to recover as many toxins as possible, we combined transcripts from all assemblers and then we used CD-HIT v. 4.7 [138] to cluster the transcripts and remove the identical transcripts. For CD-HIT, the sequence identity threshold was set to 1 and word length was set to 11. We named this method as ‘Merged’ in this study.

To evaluate each assembly with traditional metrics, and because non-toxin genes can be valuable for establishing baselines for studies of evolutionary rates and phylogenetics, we compared each assembly using two common metrics of assembly quality. We used the programme BUSCO v. 4.1.3 to identify single-copy, orthologous non-toxin loci in each assembly [139, 140]. BUSCO compares assembled contigs against lineage-specific subsets of the OrthoDB v. 10 [141] database using tBLASTn [142], followed by HMMER [143] classification of annotated contigs as follows: complete and single copy; complete and duplicated; fragmented; or missing. OrthoDB ortholog sets contain genes that exist as a single copy in the genome of 90% of the species in the database, and thus provide an evolutionary expectation of presence in an assembled gene set if the assembly is complete. For a transcriptome study, not all loci are expected to be present due to lack of expression in the target tissue, but a BUSCO analysis will permit quantitative comparison of multiple assemblies of the same transcriptome in terms of the overall number of complete and single-copy orthologous loci recovered out of the full set of loci in the OrthoDB reference database. For BUSCO analysis of the snake venom gland transcriptome assemblies, we used the Tetrapoda ortholog set containing 5310 loci. Our criterion for ranking non-toxin assembly quality is simply determining which assembler yielded the highest number of complete and single-copy matches to the OrthoDB loci.

We evaluated the quality of toxin transcript assemblies based on the quality of toxin contigs and the completeness of the final transcript sets. To determine the number of high-quality toxin transcripts assembled by each step of the assembly process, we employed a series of filtering steps to identify contigs that were (1) annotation of toxin genes and that lack (2) of signs of chimaera formation or sequence fragmentation. We used the TransDecoder tool embedded in Trinity to extract open reading frames (ORFs) from the ‘Merged’ transcripts. Then we used Blast v. 2.10.1 to do the toxin annotation.

To derive a final set of unique and high-quality toxin sequences, we applied a series of filtering criteria to our annotated toxin genes. First, we inspected the read coverage of our toxin ORFs. Since there were multiple samples, the cross contamination can happen between each other. To investigate coverage map of every ORF and saw how many reads mapped to it from each of the nine samples, we aligned our ORFs against the original reads from all nine species by BWA v. 0.7.17 [144]. We retained only those toxins that (1) had coverage > 0 across all bases in the coding region; (2) had < 100-fold coverage differentials across the length of the ORF; (3) had a coverage that varied consistently across its length (if it varied at all) since sharp discontinuities usually indicate a chimeric assembly, cross contamination, or some other problem. The final step was to manually check these remaining sequences for whether or not they really belong to the toxin family they were assigned to. For this, we manually checked those remaining toxins against sequences on GenBank using the web version of NCBI BLAST check if the annotation results from our in-house toxin database are the same as those in GenBank. We kept those toxin genes with both annotation results referred as the same toxin genes.

### Molecular phylogenetics and modelling

Protein sequences for all toxin sequences were retrieved from the UniProt database and NCBI database, then combined with the toxin transcripts from our assembly and annotation. Partial sequences and sequences with suspect assembling errors were excluded. The sequences were aligned using a combination of manual alignment of the conserved cysteine positions and alignment using the Multiple Sequence Comparison by Log-Expectation (MUSCLE) algorithm [145] implemented in AliView [146] for the blocks of sequence in between these sites. Manual refinement of the alignments was also involved because there are structural differences within different toxin families. The phylogenetic trees for different toxin families were reconstructed with MrBayes 3.2 [147] based on the amino acid sequence alignment. The output trees from MrBayes were further edited and annotated with iTol [148]. All alignments and raw tree files are available in the supplementary material. The settings for MrBayes are in Additional file 27.

Using BLAST searches, coding DNA sequences for toxin sequences were retrieved from GenBank [149] and our assembly. The sequences were trimmed to only include those codons which translate to the mature protein (signal peptide domain removed), translated, aligned, and reverse translated using AliView and the MUSCLE algorithm. Clades were designated according to the taxonomy and functional/structural difference (such as functional domains/motifs). Phylogenetic trees for each clade were generated from the resulting codon alignments using the same methods as described above. These tree topologies were used for all subsequent analyses.

The selection regime operating on a particular gene, region, or individual amino acid can be estimated by calculating the ratio of nonsynonymous nucleotide substitutions per nonsynonymous site (dN) with that of synonymous substitutions per synonymous sites (*ω* = dN/dS) for each codon in the alignment. Codons evolving with *ω* > 1 are presumed to evolve under positive selection (functional diversification), whereas *ω* < 1 indicates that the codon evolves under the influence of purifying selection. Sites with *ω* values that are near to 1 are thought to evolve neutrally. We performed a series of tests combining information from site-based and lineage-specific analyses in order to determine the most likely groups on which positive selection has been operating.

To examine the patterns of selection functioning in different toxin families, we used several of the tests for selection implemented in HyPhy v 2.220150316 beta due to their different emphases [35]. The Analyze Codon Data analysis generates overall *ω* values for an alignment, while the Fast Unconstrained Bayesian App Roximation (FUBAR) method gauges the strength of consistent positive or negative selection on individual amino acids [37]. In contrast, the Mixed Effects Model of Evolution (MEME) method identifies individual sites that were subject to episodes of positive selection in the past [36]. LRT and *P* values for MEME are found in Additional file 28.

To map residues evolving under positive selection in the three dimensional (3D) structures, custom models for each clade belonging to different toxin families were generated by representative sequences from RCSB PDB database [150] (Additional file 4). Alignments of each clade were trimmed to match these PDB structures. To view the 3D structure of the proteins, we utilised the UCSF Chimera programme v 1.10.2 with attribute files generated from FUBAR and MEME results. For FUBAR, we used the value from the beta-alpha column which is a measure of the difference between the rates of nonsynonymous (beta) and synonymous (alpha) mutations. For MEME, since MEME estimates two rates of positive selection and gives each a probability, we can take the weighted average of those two and then subtract alpha to arrive at a similar value to the one we used for FUBAR.

## Supplementary Information

**Additional file 1.** 3ftx alignment.fasta.

**Additional file 2.** 3ftx tree.tre.

**Additional file 3.** Zoomable 3ftx tree.pdf.

**Additional file 4.** Tables: Toxin types recovered for each species; Raw illumina data for each species; Statistics for each species for cleaned data; Molecular modelling templates.

**Additional file 5.** CNP alignment.fasta.

**Additional file 6.** CNP tree.tre.

**Additional file 7.** Zoomable CNP tree.pdf.

**Additional file 8.** CRISP alignment.fasta (FASTA 42 kb)

**Additional file 9.** CRISP tree.tre.

**Additional file 10.** zoomable CRISP tree.pdf.

**Additional file 11.** kallikrein alignment.fasta.

**Additional file 12.** kallikrein_tree.tre.

**Additional file 13.** zoomable kallikrein tree.pdf.

**Additional file 14.** kunitz alignment.fasta.

**Additional file 15.** kunitz tree.tre.

**Additional file 16.** zoomable kunitz tree.pdf.

**Additional file 17.** lectin alignment.fasta.

**Additional file 18.** lectin tree.tre.

**Additional file 19.** zoomable lectin tree.pdf.

**Additional file 20.** PIII SVMP alignment.fasta.

**Additional file 21.** PIII SVMP tree.tre.

**Additional file 22.** zoomalbe PIII SVMP tree.pdf.

**Additional file 23.** SVMP propeptide alignment.fasta.

**Additional file 24.** SVMP propeptide tree.tre.

**Additional file 25.** zoomable SVMP propeptide tree.pdf.

**Additional file 26.** sequences and IDs.xlsx.

**Additional file 27.** MrBayes settings.txt.

**Additional file 28.** MEME LRT_P values.csv.

## Data Availability

All data is available in the manuscript or the Additional Files. Genbank unique IDs are found in Additional File 26 (matched against assembly codes used in the manuscript for ease of cross-referencing).
