# Supplementary figures and images for: Dynamic genetic differentiation drives the widespread structural and functional convergent evolution of snake venom proteinaceous toxins
Source: BMC Biol. 2022 Jan 7;20:4. doi: 10.1186/s12915-021-01208-9 (PMC8742412; doi:10.1186/s12915-021-01208-9)

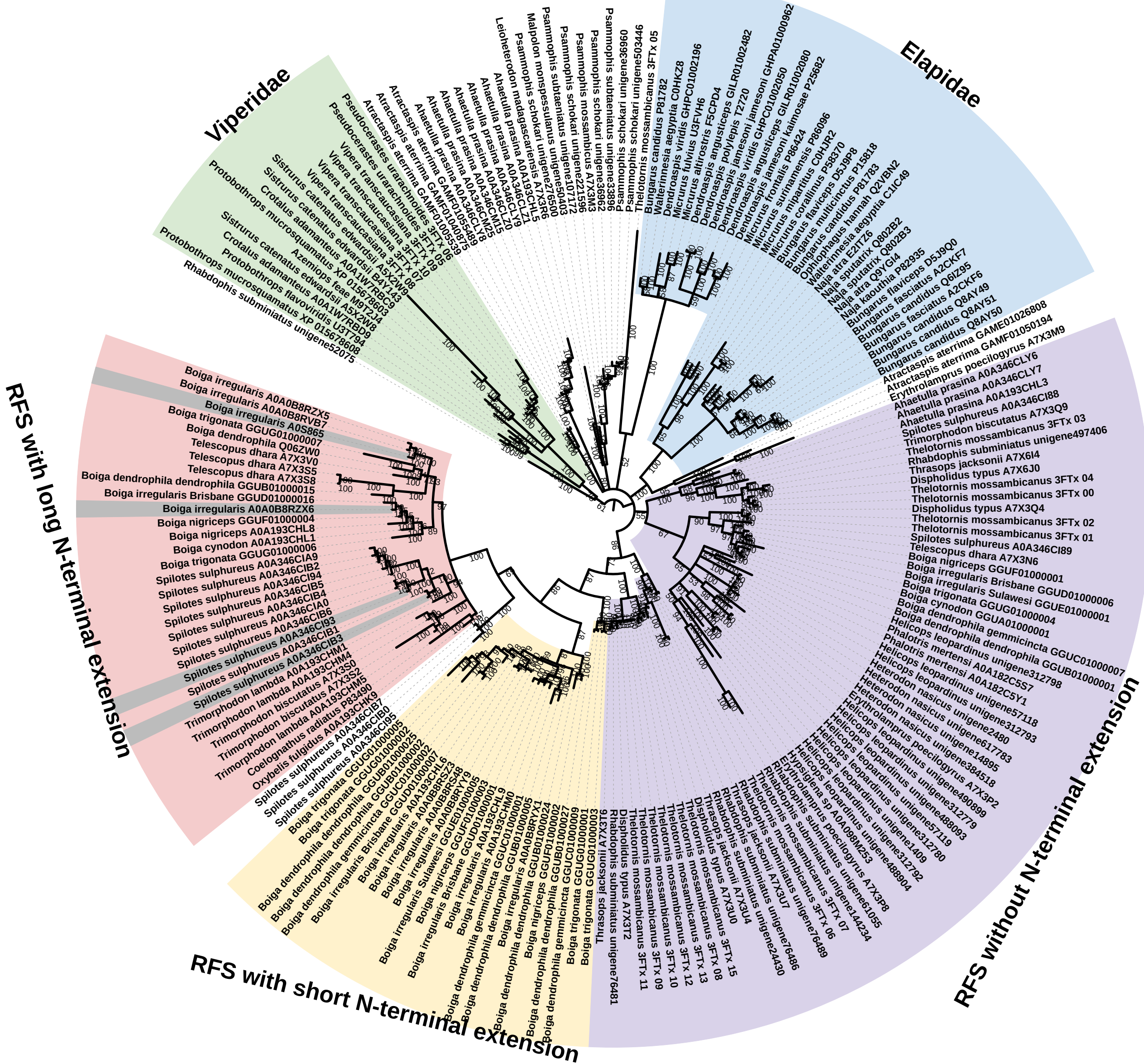

Supplement: Supplementary file 3 — Additional file 3. Zoomable 3ftx tree.pdf. [file 12915_2021_1208_MOESM3_ESM.pdf]

Tree scale: 1

Non C-terminal Tail

C-terminal Tail

C-terminal Tail

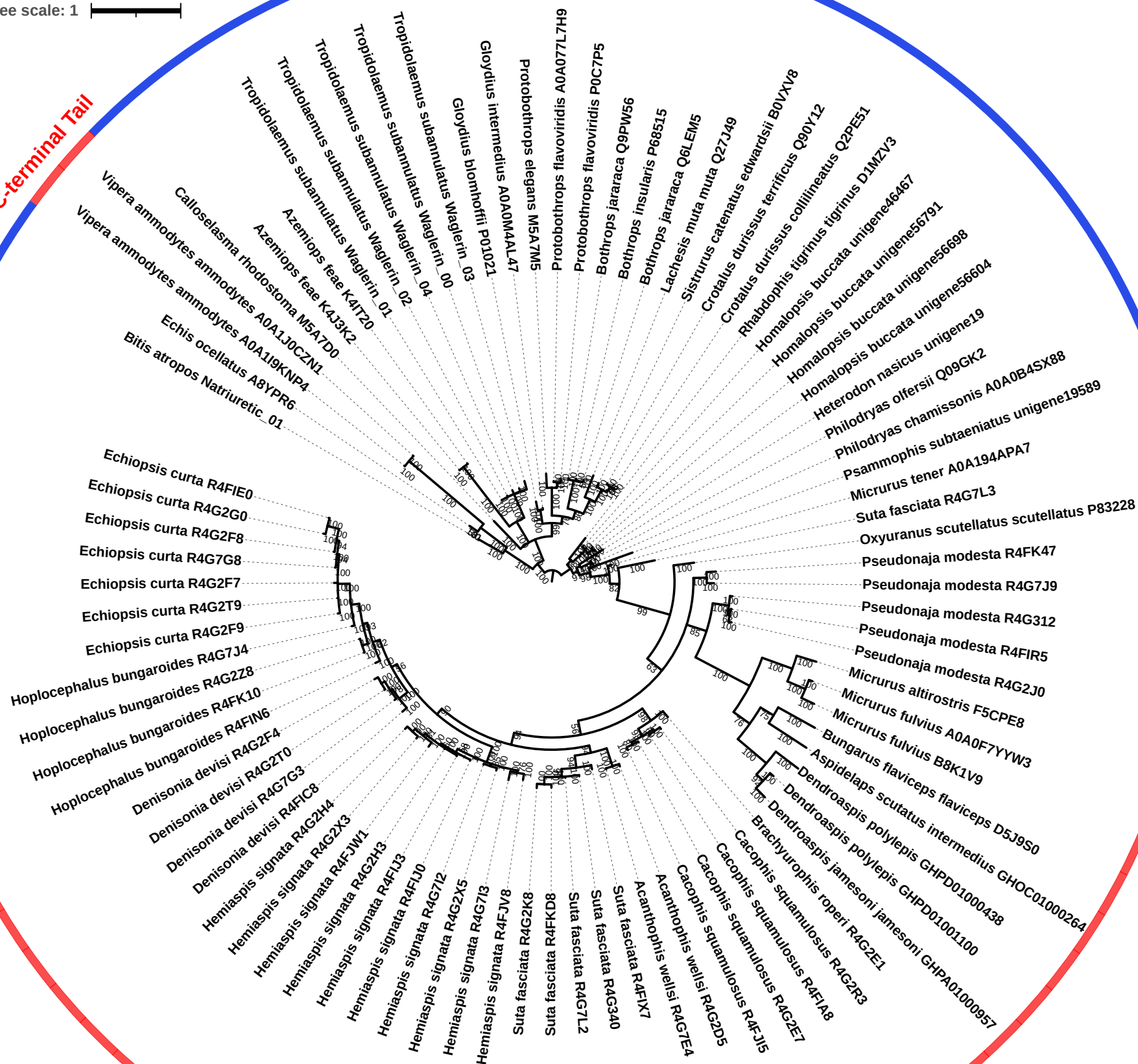

Supplement: Supplementary file 7 — Additional file 7. Zoomable CNP tree.pdf. [file 12915_2021_1208_MOESM7_ESM.pdf]

non Vipera

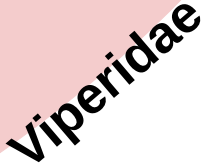

Supplement: Supplementary file 13 — Additional file 13. zoomable kallikrein tree.pdf. [file 12915_2021_1208_MOESM13_ESM.pdf]

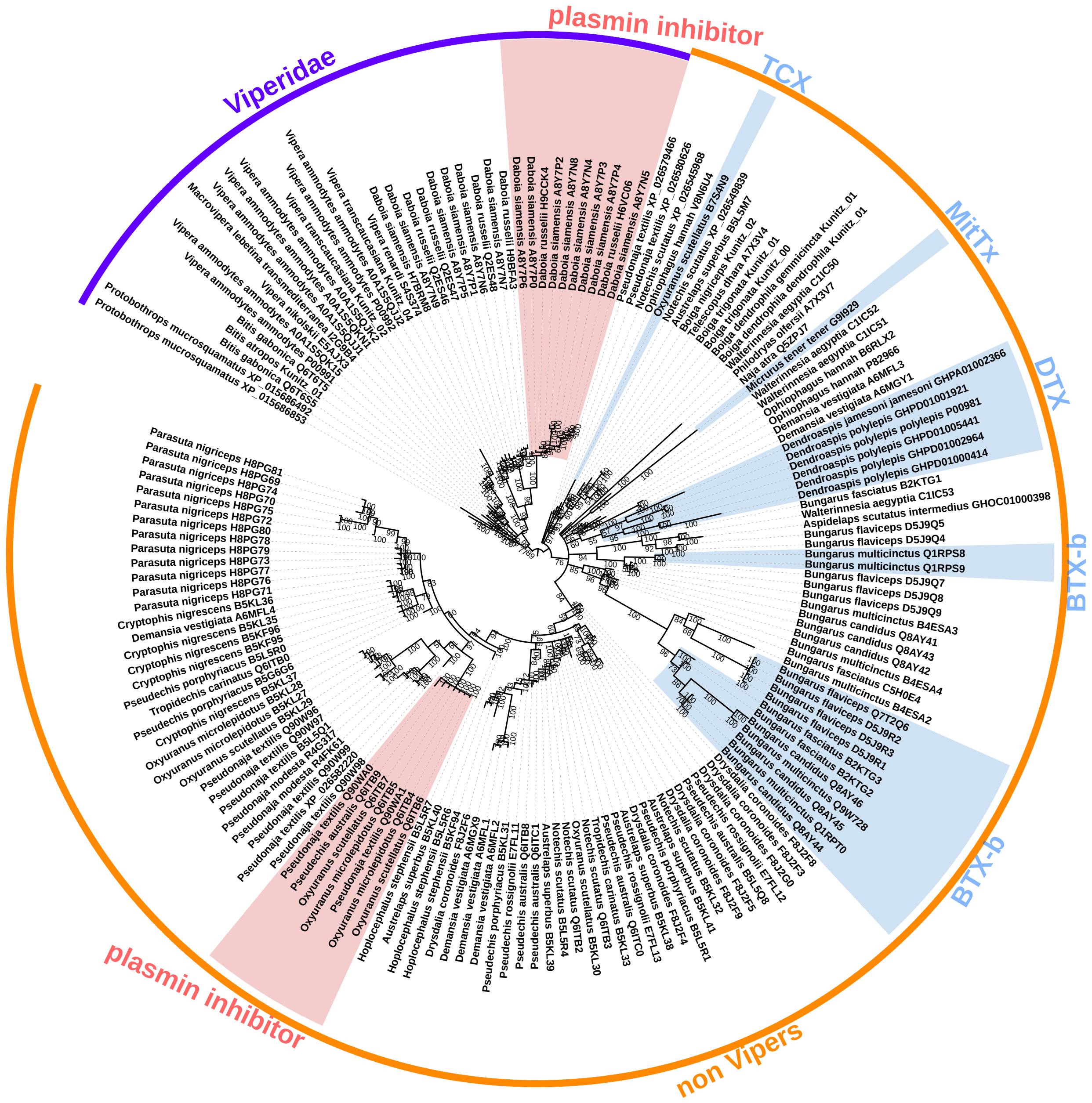

Supplement: Supplementary file 16 — Additional file 16. zoomable kunitz tree.pdf. [file 12915_2021_1208_MOESM16_ESM.pdf]

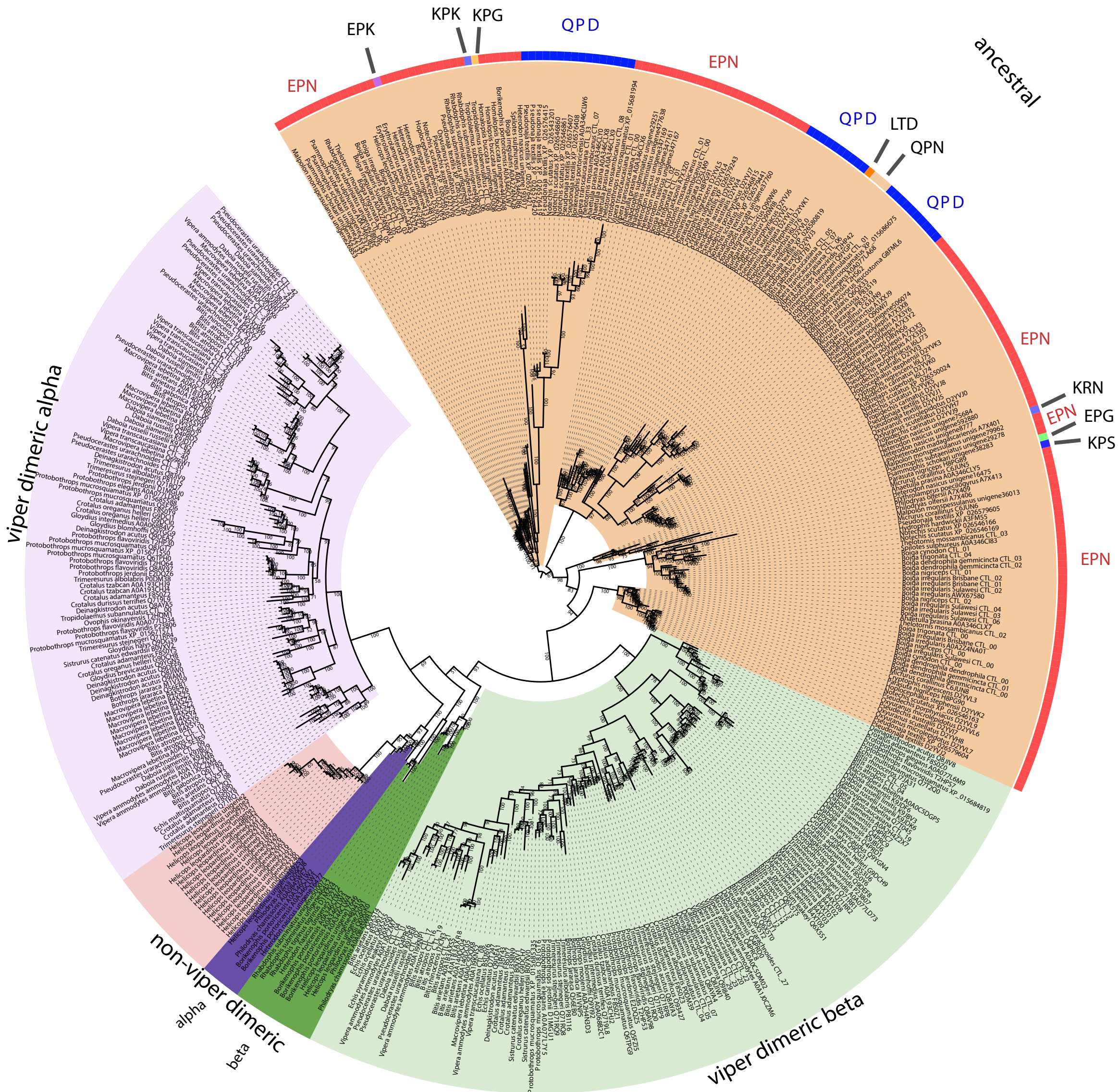

Supplement: Supplementary file 19 — Additional file 19. zoomable lectin tree.pdf. [file 12915_2021_1208_MOESM19_ESM.pdf]

### Without Cysteines

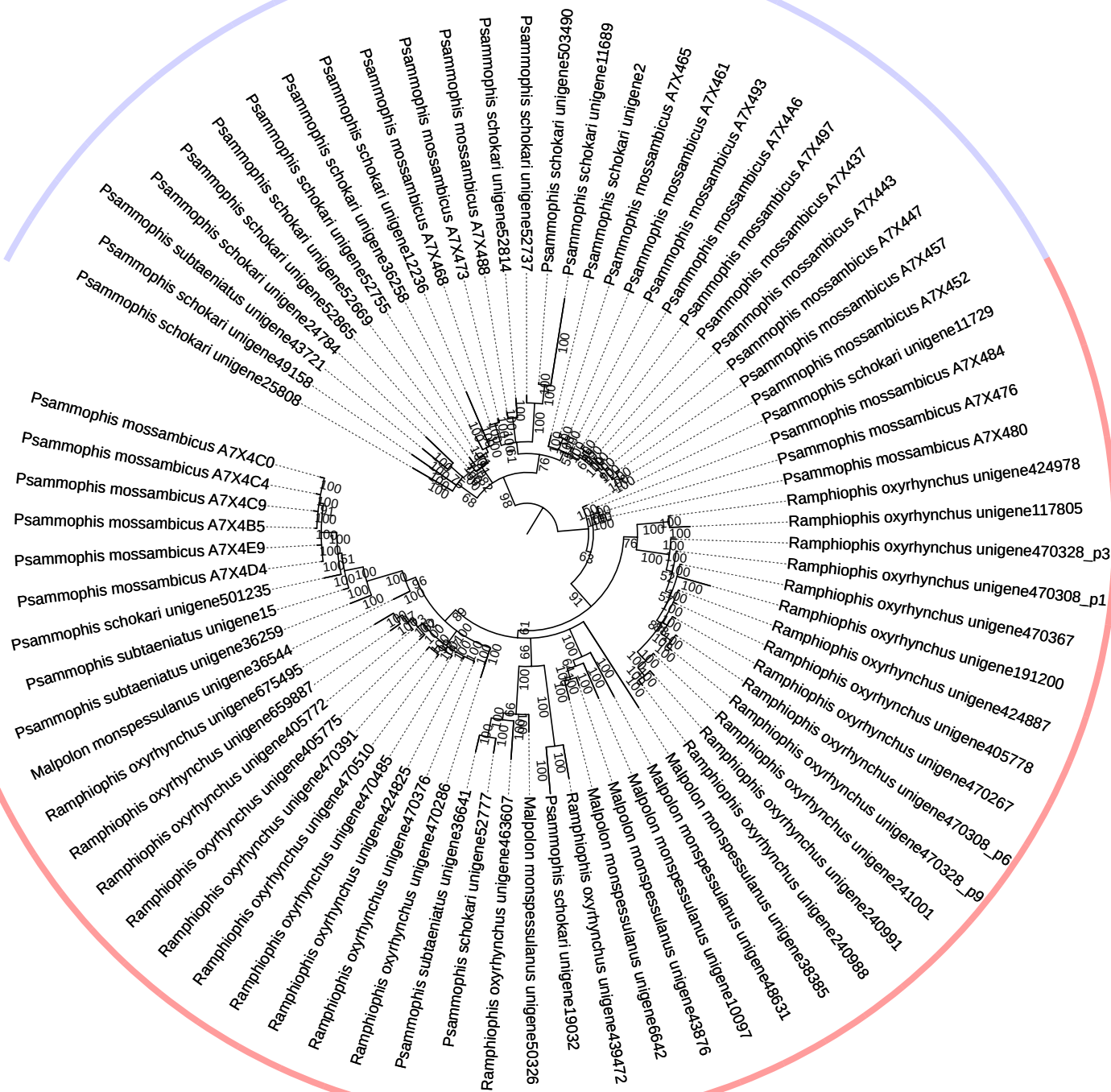

With Cysteines

Supplement: Supplementary file 25 — Additional file 25. zoomable SVMP propeptide tree.pdf. [file 12915_2021_1208_MOESM25_ESM.pdf]
