# Supplementary material for: Dynamic genetic differentiation drives the widespread structural and functional convergent evolution of snake venom proteinaceous toxins
Source: BMC Biol. 2022 Jan 7;20:4. doi: 10.1186/s12915-021-01208-9 (PMC8742412; doi:10.1186/s12915-021-01208-9)
Supplement: Supplementary file 4 — Additional file 4. Tables: Toxin types recovered for each species; Raw illumina data for each species; Statistics for each species for cleaned data; Molecular modelling templates. [file 12915_2021_1208_MOESM4_ESM.docx]

**S Table 1:** Toxin types recovered per species

|  | Homalopsidae | Colubridae | | | Lamprophiidae | | | | Viperidae | |
| --- | --- | --- | --- | --- | --- | --- | --- | --- | --- | --- |
|  | *Homalopsis buccata* | *Heterodon nasuta* | *Helicops leopardinus* | *Rhabdophis subminiatus* | *Psammophis sochureki* | *Psammophis sudanensis* | *Malpolon monspessulanus* | *Rhamphiophis oxyrhynchus* | *Pseudocerastes urarachnoides* | *Vipera transcaucasiana* |
| 3ftx |  | X | X | X | X | X | X |  | X | X |
| AChE |  | X | X | X | X |  |  |  |  |  |
| C3/CVF |  | X |  | X |  |  | X | X |  |  |
| CNP |  |  |  | X |  |  |  |  |  | X |
| CRISP |  | X | X | X | X | X | X | X |  |  |
| Cystatin | X | X | X | X |  | X | X |  | X | X |
| Extendin I |  |  |  |  |  |  |  |  |  | X |
| Factor X |  |  |  |  |  |  |  | X |  |  |
| HYAL |  |  | X |  |  |  |  | X | X |  |
| Kallikrein |  |  |  |  |  |  |  |  | X | X |
| Kunitz |  | X | X | X |  |  | X | X |  | X |
| LAAO |  |  |  |  |  |  |  |  | X | X |
| CTL | X | X | X | X |  | X | X | X | X | X |
| Lipocalin |  | X | X | X |  |  |  |  |  | X |
| NGF |  |  |  |  |  |  | X | X |  | X |
| PDE | X |  |  |  |  |  |  | X | X |  |
| PLA2 II E |  |  |  |  |  | X | X | X | X | X |
| PLB |  | X |  |  |  |  | X | X |  |  |
| Rnase | X | X |  |  | X | X |  |  | X | X |
| SVMP |  |  |  | X |  |  | X | X | X | X |
| Veficolin | X | X |  |  |  |  | X | X |  |  |
| Vespryn |  |  |  |  | X |  |  |  |  |  |
| Waprin |  |  |  |  |  |  |  | X |  |  |

**S Table 2**. Raw illumina data for each species

| Species | Total Reads (M) | Total Bases (G) | Q30(%) | GC(%) | SRA ID |
| --- | --- | --- | --- | --- | --- |
| *Helicops leopardinus* | 59.670768 | 8.950615 | 88.30 | 47.01 | SRR12802481 |
| *Rhabdophis subminiatus* | 61.908030 | 9.286205 | 87.71 | 46.84 | SRR12802480 |
| *Heterodon nasicus* | 73.443828 | 11.016574 | 88.95 | 44.21 | SRR12802479 |
| *Malpolon monspessulanus* | 59.597300 | 8.939595 | 88.54 | 46.28 | SRR12802478 |
| *Psammophis schokari* | 71.464838 | 10.719726 | 87.30 | 46.78 | SRR12802477 |
| *Psammophis subtaeniatus* | 66.870990 | 10.030648 | 88.21 | 47.21 | SRR12802476 |
| ****Rhamphiophis oxyrhynchus* | 177.324272 | 26.598641 | 87.77 | 44.67 | SRR13234020 |
| *Homalopsis buccata* | 61.067040 | 9.160056 | 88.27 | 46.76 | SRR12802475 |
| *Pseudocerastes urarachnoides* | 56.348378 | 8.452257 | 89.94 | 48.45 | SRR12802474 |
| *Vipera transcaucasiana* | 62.804494 | 9.420674 | 87.97 | 47.67 | SRR12802473 |

*Three samples were sequenced for *R. oxyrhynchus* and all raw data from these samples were combined together for further analysis

**S Table 3.** Statistics for each species for cleaned data

| Species | Total Reads (M) | Total Bases (G) | Q30(%) | GC(%) | Reads passed filters(%) |
| --- | --- | --- | --- | --- | --- |
| *Helicops leopardinus* | 57.518468 | 8.528288 | 90.05 | 46.93 | 96.39 |
| *Rhabdophis subminiatus* | 59.717436 | 8.795656 | 89.61 | 46.69 | 96.46 |
| *Heterodon nasicus* | 71.177292 | 10.574868 | 90.44 | 44.12 | 96.91 |
| *Malpolon monspessulanus* | 57.588762 | 8.533593 | 90.17 | 46.19 | 96.63 |
| *Psammophis schokari* | 68.781210 | 10.097262 | 89.56 | 46.66 | 96.24 |
| *Psammophis subtaeniatus* | 64.644424 | 9.517410 | 90.00 | 47.07 | 96.67 |
| *Rhamphiophis oxyrhynchus* | 174.167248 | 25.865585 | 88.28 | 44.56 | 98.22 |
| *Homalopsis buccata* | 58.809498 | 8.740258 | 90.02 | 46.69 | 96.30 |
| *Pseudocerastes urarachnoides* | 55.165528 | 8.150225 | 91.33 | 48.45 | 97.90 |
| *Vipera transcaucasiana* | 60.575680 | 8.951936 | 89.81 | 47.67 | 96.45 |

**S Table 4:** Molecular modelling templates

|  | **Clades** | **Sequence for 3D modelling** | **PDB ID** |
| --- | --- | --- | --- |
| **3ftx** | Elapidae | *Micrurus altirostris* F5CPD4 | 1ijc |
|  | Viperidae | *Sistrurus catenatus edwardsii* A5X2W8 | 3neq |
|  | RFS with N-terminal Extension | *Oxybelis fulgidus* A0A193CHK9 | 2h7z |
|  | RFS with short N-terminal Extension | *Boiga irregularis* A0A0B8RYY9 | 2h7z |
|  | RFS with long N-terminal Extension | *Boiga irregularis* A0S865 | 2h7z |
|  | RFS without N-terminal Extension | *Leioheterodon madagascariensis* A7X3R6 | 6wjc |
| **CRiSP** | Elapid type | *Pseudechis australis* AAL65291 | 2dda |
|  | Viperid type | *Naja atra* AAP85301 | 3mz8 |
|  | RFS type | *Echis coloratus* A0A0A1WD40 | 1wvr |
| **Kallikrein** | non Vipers | *Philodryas olfersii* Q09GK1 | 2aip |
|  | Viperidae | *Gloydius halys* AFM29142 | 4e7n |
| **Kunitz** | Viperidae | *Vipera ammodytes* P00992 | 6a5i |
|  | DTX | *Dendroaspis polylepis* P00981 | 1dtk |
|  | BTX | *Bungarus multicinctus* Q1RPT0 | 1bun |
|  | non vipers | *Bungarus multicinctus* Q1RPT0 | 1bun |
|  | Viper type plasmin inhibitor | *Vipera ammodytes* P00992 | 6a5i |
|  | non-viper type plasmin inhibitor | *Pseudonaja textilis* Q90WA1 | 5zj3 |
| **C-type Lectin** | ancestral | *Erythrolamprus poecilogyrus* A7X3Z7 | 5f2q |
|  | viper dimeric alpha | *Bothrops jararaca* Q56EB1 | 5f2q |
|  | non-viper dimeric | *Philodryas olfersii* Q09GK0 | 1v7p |
|  | viper dimeric beta | *Echis multisquamatus* Q7T2Q0 | 1fvu |
| **SVMP PIII** | Colubridae | *Cerberus rynchops* D8VNS0 | 2dw2 |
|  | Elapidae | *Naja atra* D5LMJ3 | 3k7l |
|  | Viperidae | *Trimeresurus stejnegeri* Q2LD49 | 3k7l |
