# Supplementary material for: Dynamic genetic differentiation drives the widespread structural and functional convergent evolution of snake venom proteinaceous toxins
Source: BMC Biol. 2022 Jan 7;20:4. doi: 10.1186/s12915-021-01208-9 (PMC8742412; doi:10.1186/s12915-021-01208-9)
Supplement: Supplementary file 10 — Additional file 10. zoomable CRISP tree.pdf. [file 12915_2021_1208_MOESM10_ESM.pdf]

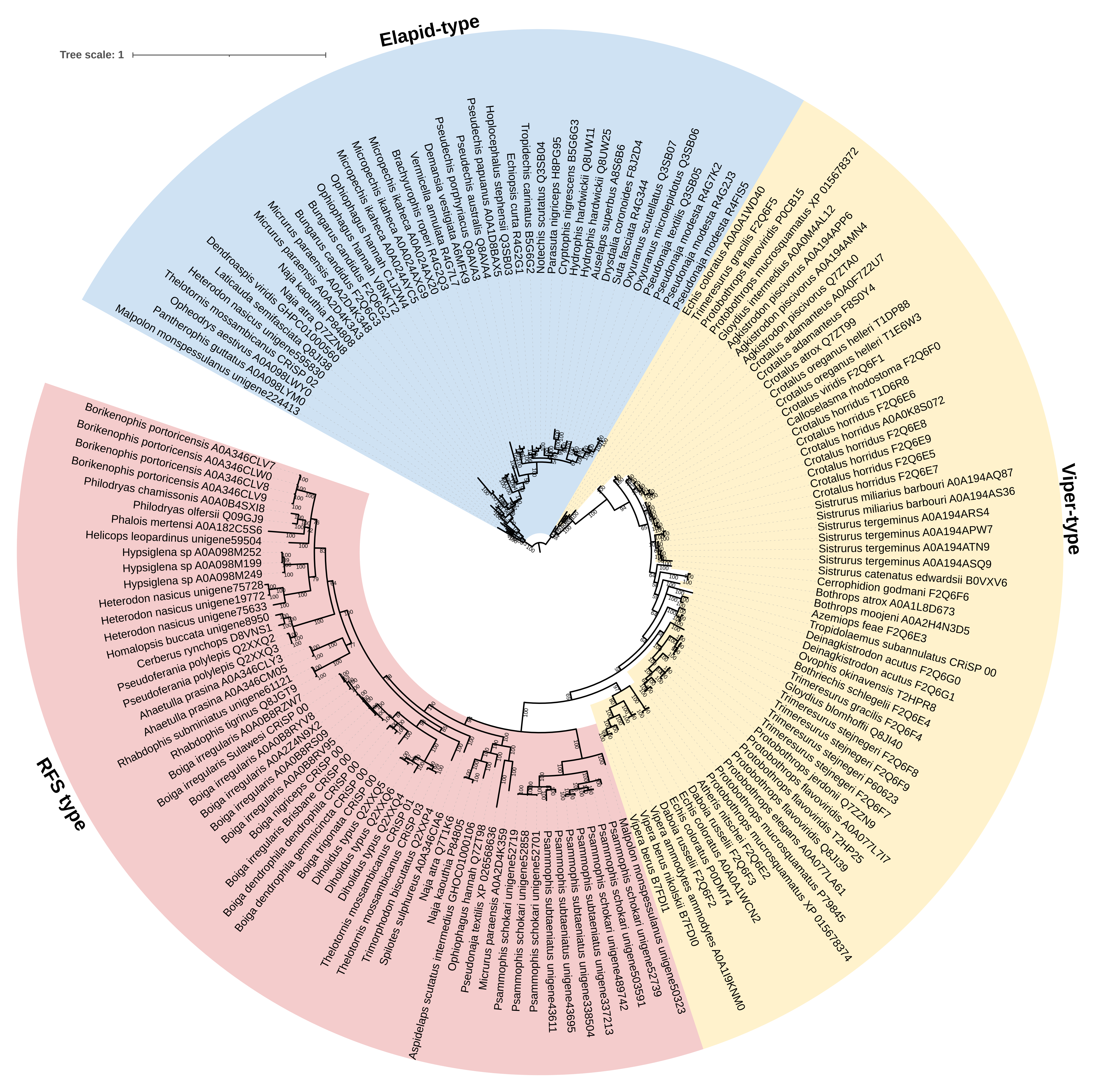

Tree scale: 1
Elapid-type
Malpolon monspessulanus unigene224413
Pantherophis guttatus A0A098LYM0
Opheodrys aestivus A0A098LWY0
Thelotornis mossambicanus CRiSP 02
Heterodon nasicus unigene595830
Laticauda semifasciata Q8JI38
Dendroaspis viridis GHPC01000560
Naja atra Q7ZZN8
Naja kaouthia P84808
Micrurus paraensis A0A2D4K3A3
Micrurus paraensis A0A2D4K348
Bungarus candidus F2Q6G3
Bungarus candidus F2Q6G2
Ophiophagus hannah V8NKT2
Ophiophagus hannah C1JZW4
Micropechis ikaheca A0A024AYC5
Micropechis ikaheca A0A024AXG9
Brachyurophis roperi R4G2Q3
Vermicella annulata R4G7L7
Demansia vestigiata A6MFK9
Pseudechis porphyriacus Q8AVA3
Pseudechis australis Q8AVA4
Pseudechis papuanus A0A1D8BAX5
Hoplocephalus stephensii Q3SB03
Echiopsis curta R4G2G1
Tropidechis carinatus B5G6G2
Notechis scutatus Q3SB04
Parasuta nigriceps H8PG95
Cryptophis nigrescens B5G6G3
Hydrophis hardwickii Q8UW11
Hydrophis hardwickii Q8UW25
Auselaps superbus A8S6B6
Drysdalia coronoides F8J2D4
Suta fasciata R4G344
Oxyuranus scutellatus Q3SB07
Oxyuranus microlepidotus Q3SB06
Pseudonaja textilis Q3SB05
Pseudonaja modesta R4G7K2
Pseudonaja modesta R4G2J3
Pseudonaja modesta R4FIS5
Viper-type
Echis coloratus A0A0A1WD40
Trimeresurus gracilis F2Q6F5
Protobothrops flavoviridis P0CB15
Protobothrops mucrosquamatus XP 015678372
Gloydius intermedius A0A0M4AL12
Agkistrodon piscivorus A0A194APP6
Agkistrodon piscivorus A0A194AMN4
Agkistrodon piscivorus Q7ZTA0
Crotalus adamanteus A0A0F7Z2U7
Crotalus adamanteus F8S0Y4
Crotalus atrox Q7ZT99
Crotalus oreganus helleri T1DP88
Crotalus oreganus helleri T1E6W3
Crotalus viridis F2Q6F1
Calloselasma rhodostoma F2Q6F0
Crotalus horridus T1D6R8
Crotalus horridus F2Q6E6
Crotalus horridus A0A0K8S072
Crotalus horridus F2Q6E8
Crotalus horridus F2Q6E9
Crotalus horridus F2Q6E5
Crotalus horridus F2Q6E7
Sistrurus miliarius barbouri A0A194AQ87
Sistrurus miliarius barbouri A0A194AS36
Sistrurus tergeminus A0A194ARS4
Sistrurus tergeminus A0A194APW7
Sistrurus tergeminus A0A194ATN9
Sistrurus tergeminus A0A194ASQ9
Sistrurus catenatus edwardsii B0VXV6
Cerrophidion godmani F2Q6F6
Bothrops atrox A0A1L8D673
Bothrops moojeni A0A2H4N3D5
Azemiops feae F2Q6E3
Tropidolaemus subannulatus CRiSP 00
Deinagkistrodon acutus F2Q6G0
Deinagkistrodon acutus F2Q6G1
Ovophis okinavensis T2HPR8
Bothriechis schlegelii F2Q6E4
Trimeresurus gracilis F2Q6F4
Gloydius blomhoffii Q8JI40
Trimeresurus stejnegeri F2Q6F8
Trimeresurus stejnegeri F2Q6F9
Trimeresurus stejnegeri P60623
Trimeresurus jerdonii Q7ZZN9
Trimeresurus stejnegeri F2Q6F7
Protobothrops flavoviridis A0A077L7I7
Protobothrops flavoviridis T2HP25
Protobothrops flavoviridis Q8JI39
Protobothrops elegans A0A077LA61
Protobothrops mucrosquamatus P79845
Protobothrops mucrosquamatus XP 015678374
Atheris nitschei F2Q6E2
Daboia russelii F2Q6F3
Echis coloratus A0A0A1WCN2
Echis coloratus P0DMT4
Daboia russelii F2Q6F2
Vipera ammodytes ammodytes A0A1I9KNM0
Vipera berus nikolskii B7FDI0
Vipera berus B7FDI1
RFS type
Malpolon monspessulanus unigene50323
Psammophis schokari unigene52739
Psammophis schokari unigene503591
Psammophis schokari unigene489742
Psammophis subtaeniatus unigene337213
Psammophis subtaeniatus unigene338504
Psammophis subtaeniatus unigene43695
Psammophis subtaeniatus unigene43611
Psammophis schokari unigene52701
Psammophis schokari unigene52858
Psammophis schokari unigene52719
Micrurus paraensis A0A2D4K359
Pseudonaja textilis XP 026568636
Ophiophagus hannah Q7ZT98
Aspidelaps scutatus intermedius GHOC01000106
Naja kaouthia P84805
Naja atra Q7T1K6
Spilotes sulphureus A0A346CIA6
Trimorphodon biscutatus Q2XXP4
Thelotornis mossambicanus CRISP 03
Thelotornis mossambicanus CRISP 01
Diholidus typus Q2XXQ4
Diholidus typus Q2XXQ6
Diholidus typus Q2XXQ5
Boiga trigonata CRiSP 00
Boiga dendrophila gemmicincta CRiSP 00
Boiga dendrophila dendrophila CRiSP 00
Boiga irregularis Brisbane CRiSP 00
Boiga nigriceps CRiSP 00
Boiga irregularis A0A0B8RV95
Boiga irregularis A0A0B8RS09
Boiga irregularis A0A2Z4N9X2
Boiga irregularis A0A0B8RYV8
Boiga irregularis Sulawesi CRiSP 00
Boiga irregularis A0A0B8RZW7
Rhabdophis tigrinus Q8JGT9
Rhabdophis subminiatus unigene61121
Ahaetulla prasina A0A346CM05
Ahaetulla prasina A0A346CLY3
Pseudoferania polylepis Q2XXQ3
Pseudoferania polylepis Q2XXQ2
Cerberus rynchops D8VNS1
Homalopsis buccata unigene8950
Heterodon nasicus unigene75633
Heterodon nasicus unigene19772
Heterodon nasicus unigene75728
Hypsiglena sp A0A098M249
Hypsiglena sp A0A098M199
Hypsiglena sp A0A098M252
Helicops leopardinus unigene59504
Phalois mertensi A0A182C5S6
Philodryas olfersii Q09GJ9
Philodryas chamissonis A0A0B4SXI8
Borikenophis portoricensis A0A346CLV9
Borikenophis portoricensis A0A346CLV8
Borikenophis portoricensis A0A346CLW0
Borikenophis portoricensis A0A346CLV7
