# Supplementary material for: Dynamic genetic differentiation drives the widespread structural and functional convergent evolution of snake venom proteinaceous toxins
Source: BMC Biol. 2022 Jan 7;20:4. doi: 10.1186/s12915-021-01208-9 (PMC8742412; doi:10.1186/s12915-021-01208-9)
Supplement: Supplementary file 22 — Additional file 22. zoomalbe PIII SVMP tree.pdf. [file 12915_2021_1208_MOESM22_ESM.pdf]

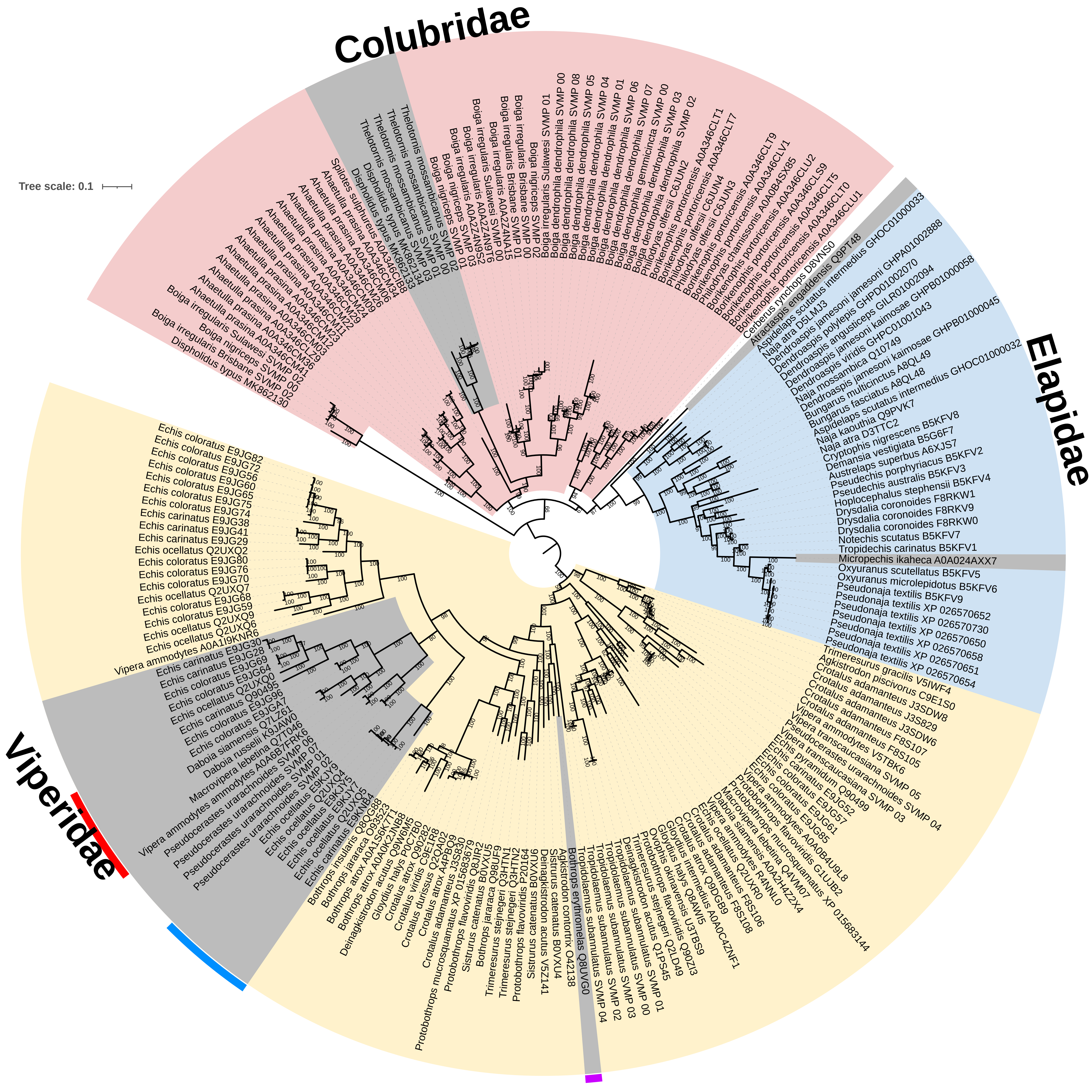
Colubridae
Elapidae
Viperidae
Tree scale: 0.1
Dispholidus typus MK862130
Boiga irregularis Brisbane SVMP 02
Boiga nigriceps SVMP 00
Boiga irregularis Sulawesi SVMP 02
Ahaetulla prasina A0A346CM41
Ahaetulla prasina A0A346CM36
Ahaetulla prasina A0A346CLZ9
Ahaetulla prasina A0A346CM03
Ahaetulla prasina A0A346CM23
Ahaetulla prasina A0A346CM12
Ahaetulla prasina A0A346CM11
Ahaetulla prasina A0A346CM22
Ahaetulla prasina A0A346CM24
Ahaetulla prasina A0A346CM26
Ahaetulla prasina A0A346CM06
Ahaetulla prasina A0A346CM09
Spilotes sulphureus A0A346CIB8
Dispholidus typus MK862134
Dispholidus typus MK862133
Thelotornis mossambicanus SVMP 03
Thelotornis mossambicanus SVMP 01
Thelotornis mossambicanus SVMP 00
Thelotornis mossambicanus SVMP 02
Boiga nigriceps SVMP 01
Boiga nigriceps SVMP 03
Boiga irregularis A0A2Z4N9S2
Boiga irregularis Sulawesi SVMP 00
Boiga irregularis A0A2Z4N9T6
Boiga irregularis A0A2Z4NA15
Boiga irregularis Brisbane SVMP 00
Boiga irregularis Brisbane SVMP 01
Boiga nigriceps SVMP 02
Boiga irregularis Sulawesi SVMP 01
Boiga dendrophila SVMP 00
Boiga dendrophila dendrophila SVMP 08
Boiga dendrophila dendrophila SVMP 05
Boiga dendrophila dendrophila SVMP 04
Boiga dendrophila dendrophila SVMP 01
Boiga dendrophila dendrophila SVMP 06
Boiga dendrophila gemmicincta SVMP 07
Boiga dendrophila gemmicincta SVMP 00
Boiga dendrophila dendrophila SVMP 03
Boiga dendrophila dendrophila SVMP 02
Philodryas olfersii C6JUN2
Borikenophis portoricensis A0A346CLT1
Philodryas olfersii C6JUN4
Borikenophis portoricensis A0A346CLT7
Philodryas chamissonis C6JUN3
Borikenophis portoricensis A0A346CLT9
Philodryas chamissonis A0A0B4SX85
Borikenophis portoricensis A0A346CLV1
Borikenophis portoricensis A0A346CLU2
Borikenophis portoricensis A0A346CLS9
Borikenophis portoricensis A0A346CLT5
Borikenophis portoricensis A0A346CLT0
Borikenophis portoricensis A0A346CLU1
Cerberus rynchops D8VNS0
Atractaspis engaddensis Q9PT48
Aspidelaps scutatus intermedius GHOC01000033
Naja atra D5LMJ3
Dendroaspis jamesoni jamesoni GHPA01002888
Dendroaspis polylepis GHPD01002070
Dendroaspis angusticeps GILR01002094
Dendroaspis jamesoni kaimosae GHPB01000058
Dendroaspis viridis GHPC01001043
Naja mossambica Q10749
Dendroaspis jamesoni kaimosae GHPB01000045
Bungarus multicinctus A8QL49
Bungarus fasciatus A8QL48
Aspidelaps scutatus intermedius GHOC01000032
Naja kaouthia Q9PVK7
Naja atra D3TTC2
Cryptophis nigrescens B5KFV8
Demansia vestigiata B5G6F7
Austrelaps superbus A6XJS7
Pseudechis porphyriacus B5KFV2
Pseudechis australis B5KFV3
Hoplocephalus stephensii B5KFV4
Drysdalia coronoides F8RKW1
Drysdalia coronoides F8RKV9
Drysdalia coronoides F8RKW0
Notechis scutatus B5KFV7
Tropidechis carinatus B5KFV1
Micropechis ikaheca A0A024AXX7
Oxyuranus scutellatus B5KFV5
Oxyuranus microlepidotus B5KFV6
Pseudonaja textilis B5KFV9
Pseudonaja textilis XP 026570652
Pseudonaja textilis XP 026570730
Pseudonaja textilis XP 026570650
Pseudonaja textilis XP 026570658
Pseudonaja textilis XP 026570651
Pseudonaja textilis XP 026570654
Trimeresurus gracilis V5IWF4
Agkistrodon piscivorus C9E1S0
Crotalus adamanteus J3SDW8
Crotalus adamanteus J3S829
Crotalus adamanteus J3SDW6
Crotalus adamanteus F8S107
Crotalus adamanteus F8S105
Vipera ammodytes V5TBK6
Vipera transcaucasiana SVMP 05
Pseudocerastes urarachnoides SVMP 04
Vipera transcaucasiana SVMP 03
Macrovipera lebetina Q90499
Echis pyramidum Q90499
Echis coloratus E9JG52
Echis coloratus E9JG57
Echis carinatus E9JG61
Echis coloratus E9JG85
Vipera ammodytes A0A0B4U9L8
Protobothrops flavoviridis G1UJB2
Protobothrops mucrosquamatus XP 015683144
Vipera lebetina Q4VM07
Macrovipera lebetina A0A2H4Z2X4
Vipera ammodytes R4NNL0
Daboia siamensis Q2UXR0
Crotalus adamanteus F8S106
Echis ocellatus A0A0C4ZNF1
Crotalus adamanteus F8S108
Crotalus intermedius Q8AWI5
Crotalus atrox Q9DGB9
Gloydius halys U3TBS9
Gloydius okinavensis Q90ZI3
Ovophis okinavensis Q90ZI3
Trimeresurus flavoviridis Q2LD49
Protobothrops stejnegeri Q1PS45
Deinagkistrodon acutus SVMP 01
Tropidolaemus subannulatus SVMP 00
Tropidolaemus subannulatus SVMP 03
Tropidolaemus subannulatus SVMP 02
Tropidolaemus subannulatus SVMP 04
Bothrops erythromelas Q8UVG0
Agkistrodon contortrix O42138
Sistrurus catenatus B0VXU4
Deinagkistrodon acutus V5Z141
Sistrurus catenatus B0VXU6
Protobothrops flavoviridis P20164
Trimeresurus stejnegeri Q3HTN2
Trimeresurus stejnegeri Q3HTN1
Bothrops jararaca Q98UF9
Sistrurus catenatus B0VXU5
Protobothrops flavoviridis Q8JIR2
Protobothrops mucrosquamatus XP 015683679
Crotalus adamanteus J3S830
Crotalus atrox A4PBQ9
Crotalus durissus Q2QA02
Crotalus viridis C9E1R8
Crotalus atrox Q90282
Gloydius halys P0C7B0
Deinagkistrodon acutus Q9W6M5
Bothrops atrox A0A0K2JNB8
Bothrops atrox A0A1S6K7T1
Bothrops jararaca O93523
Bothrops insularis Q8QG88
Echis carinatus E9KJB4
Echis ocellatus Q2UXQ5
Echis ocellatus E9KJY7
Echis ocellatus Q2UXQ4
Echis ocellatus E9KJY9
Pseudocerastes urarachnoides SVMP 01
Pseudocerastes urarachnoides SVMP 02
Pseudocerastes urarachnoides SVMP 07
Pseudocerastes urarachnoides SVMP 06
Vipera ammodytes A0A6B7FRK6
Macrovipera lebetina Q7T046
Daboia russelii K9JAW0
Daboia siamensis Q7LZ61
Echis coloratus E9JGA7
Echis coloratus E9JG96
Echis carinatus Q90495
Echis ocellatus Q2UXQ0
Echis coloratus E9JG64
Echis coloratus E9JG69
Echis carinatus E9JG28
Echis carinatus E9JG30
Vipera ammodytes A0A1I9KNR6
Echis ocellatus Q2UXQ6
Echis ocellatus Q2UXQ9
Echis coloratus E9JG59
Echis coloratus E9JG68
Echis ocellatus Q2UXQ7
Echis coloratus E9JG70
Echis coloratus E9JG76
Echis coloratus E9JG80
Echis ocellatus Q2UXQ2
Echis carinatus E9JG29
Echis carinatus E9JG41
Echis carinatus E9JG38
Echis coloratus E9JG74
Echis coloratus E9JG75
Echis coloratus E9JG65
Echis coloratus E9JG60
Echis coloratus E9JG56
Echis coloratus E9JG72
Echis coloratus E9JG82
